# Bioinspired Electrolyte-Gated Organic Synaptic Transistors: From Fundamental Requirements to Applications

**DOI:** 10.1007/s40820-025-01708-1

**Published:** 2025-03-24

**Authors:** Yuanying Liang, Hangyu Li, Hu Tang, Chunyang Zhang, Dong Men, Dirk Mayer

**Affiliations:** 1 Guangdong Artificial Intelligence and Digital Economy Laboratory (Guangzhou), Guangzhou, 510335 People’s Republic of China; 2 Institute of Biological Information Processing, Bioelectronics IBI-3, Forschungszentrum Jülich, 52425 Jülich, Germany; 3 Guangzhou Liby Group Co., Ltd, Guangzhou, 510370 People’s Republic of China; 4 Guangzhou National Laboratory, Guangzhou, 510005 People’s Republic of China

**Keywords:** Neuromorphic device, Tunable synaptic plasticity, Electrolyte-gated organic transistors, Neurochemical signals, Artificial perception systems

## Abstract

Rapid development of artificial intelligence requires the implementation of hardware systems with bioinspired parallel information processing and presentation and energy efficiency. Electrolyte-gated organic transistors (EGOTs) offer significant advantages as neuromorphic devices due to their ultra-low operation voltages, minimal hardwired connectivity, and similar operation environment as electrophysiology. Meanwhile, ionic–electronic coupling and the relatively low elastic moduli of organic channel materials make EGOTs suitable for interfacing with biology. This review presents an overview of the device architectures based on organic electrochemical transistors and organic field-effect transistors. Furthermore, we review the requirements of low energy consumption and tunable synaptic plasticity of EGOTs in emulating biological synapses and how they are affected by the organic materials, electrolyte, architecture, and operation mechanism. In addition, we summarize the basic operation principle of biological sensory systems and the recent progress of EGOTs as a building block in artificial systems. Finally, the current challenges and future development of the organic neuromorphic devices are discussed.

## Highlights

- Neuromorphic device with bioinspired parallel information processing and presentation and energy efficiency is desired for the rapid development of artificial intelligence.
- Electrolyte-gated organic transistors can be leveraged to emulate versatile synaptic functions with tunable properties and excellent biocompatibility.
- Recent development regarding the organic channel materials, neuromorphic device handling neurochemical signals, the basic requirements to achieve artificial synapse, and the applications on mimicking perception functions are reviewed.

## Graphical abstract

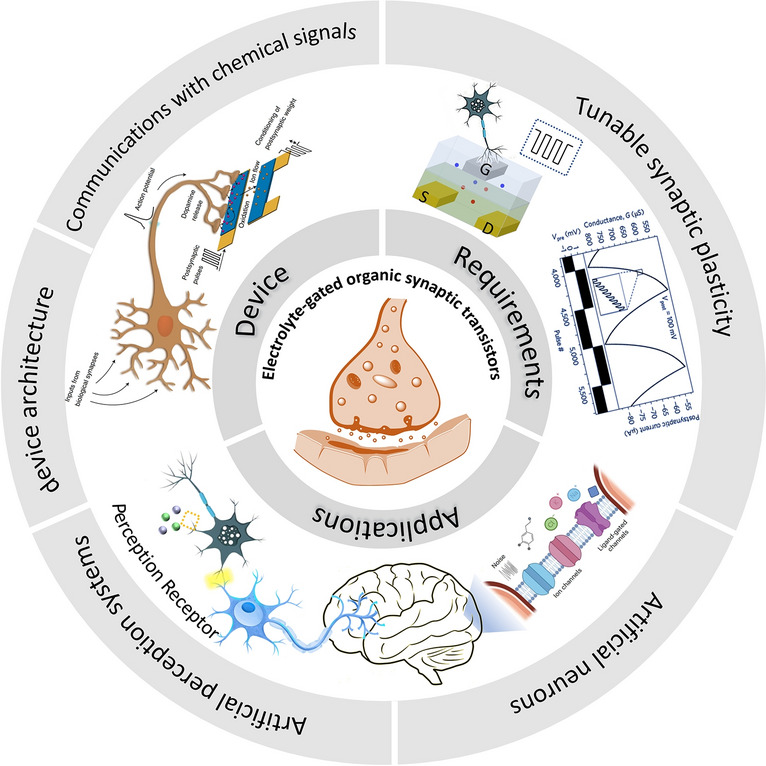

## Introduction

The fulminant development of digitization has placed great demands on the processing and presentation of information. Current digital logic computation relies heavily on the von Neumann computer architecture with separate processing and memory elements, which, however, is inadequate for future artificial intelligence due to its high energy consumption and limited parallel computing capability [1]. The biological neural networks in a human brain consist of ~ 10^12^ neurons with ~ 10^15^ interconnecting synapses. These networks can efficiently execute massive parallel information processing and simultaneously perform storage tasks with ultra-low power consumption (10 fJ/synaptic event) and fault-tolerant characteristics [2]. Since information processing and transmission between neurons rely on electrical/chemical signals mediated by ions and neurotransmitters released at the synapse in a complex network [3, 4], the development of synaptic devices with integrated signal transduction and neuron-inspired signal processing functions is highly desired to emulate the functions of biological neurons and synapses toward the upcoming artificial intelligence era [5–8].

The ideal bioinspired synaptic devices generally require: (1) low power consumption, which is the key advantages compared to the conventional von Neumann computer architecture, (2) long state retention time and stability at a given state, ensuring long-term data storage, especially for write-once–read-many times devices [9–12], and (3) ability to emulate specific typical brain-like functions, such as short-/long-term plasticity, excitatory postsynaptic current (EPSC), paired-pulse facilitation (PPF), and filtering characteristics. In recent decades, intense scientific efforts have focus on the realization of bioinspired (or neuromorphic) devices with adaptive properties to emulate the basic synaptic functions of neuromorphic computation and memory [7]. Several conceptual neuromorphic devices have been proposed including two-terminal memristors with metal–insulator–metal configuration [13–17] and three-terminal transistors [18]. Unlike the two-terminal memristive devices, which require separate circuits for each input, the structural nature of synaptic transistors with physically separated input and output terminals potentially allows information processing and learning to occur synchronously [19], i.e., the synaptic weight of each gate input can be tuned individually during fabrication [19, 20].

Since synaptic functions were first mimicked by Mead in 1996 using a floating-gate silicon metal–oxide semiconductor transistor, tremendous efforts have been devoted to investigate new device concepts, active materials, and switching mechanisms to improve device performance. The key parameters that determine the transistor performance are the channel material and the gate dielectric layer. Organic electronic materials, especially the organic mixed ionic–electronic conductors (OMIECs), cover a wide spectrum of properties including the structural relatedness to many biological compounds, low-cost solution preparation processes, and easy modification of their chemical, electrical, and mechanical properties for desired applications, making them ideal candidates for neuromorphic devices [21–23]. The communications between neurons through synapse are based on ion- or biochemical-modulated dynamic process [24]. Most of the neuromorphic functions achieved so far in solid-state devices suffer from the complicated integration of large numbers of transistors and passive electronic components, thus resulting in bulky biomimetic circuits, that are not suitable for direct biointerfacing. In contrast, electrolyte-gated organic transistors (EGOTs) enable neuromorphic functions in an aqueous environment, which not only provides superior compatibility with biological systems but also provides access to various chemical processes for the implementation of multiple functions to the neuromorphic devices. The electrolyte-gated transistors (EGTs) exert their versatile potentials as promising candidates for neuromorphic devices due to their low operation voltages and their ability to transduce and amplify small biological signals into electronic signals [25–29]. The channel conductivity in EGTs is modulated by ionic migration between electrolyte and organic channel under the application of gate bias, which facilitates the strong coupling between electronic and ionic charge carriers and enables the efficient communication between electronic devices and biology [30–32]. The core of the neuromorphic functionality is the variation of the reversible or irreversible variation of the channel conductance as a function of external stimuli (electrical, electrophysiological, or biochemical).

**Fig. 1:**
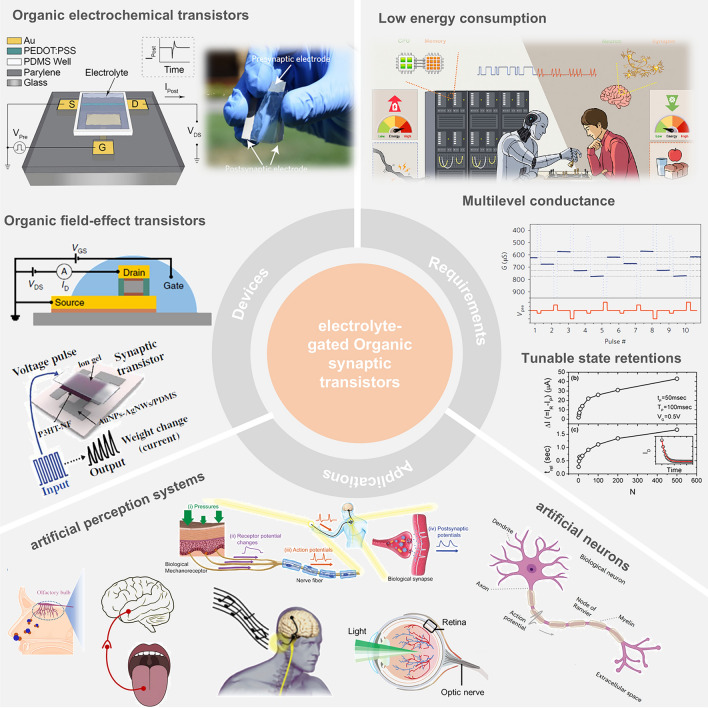
Electrolyte-gated organic synaptic transistors (EGOST). Left: representative organic synaptic devices based on organic electrochemical transistors and organic field-effect transistors. Images taken from the following references: [32] Copyright 2015, Wiley–VCH; [33] Copyright 2017, Springer Nature; [34] Copyright 2019, Springer Nature; [35] Copyright 2019, AAAS. Right: Basic requirements to achieve high-performance artificial synapse. Images produced with permission from references: [19] Copyright 2021, Cell Press; [33] Copyright 2017, Springer Nature; [36] Copyright 2015, AIP Publishing. Bottom: Applications of electrolyte-gated organic synaptic transistors. Reproduced with permission. [37] Copyright 2023, Springer Nature; [38] Copyright 2019, Elsevier; [39] Copyright 2022, Wiley–VCH; [40] Copyright 2024, Wiley–VCH; [41] Copyright 2018, AAAS

In this review, we summarize the recent progress on bioinspired electrolyte-gated organic synaptic transistors with the aim of investigating the fundamental working mechanism of the corresponding transistor devices. The basic requirements to achieve an artificial synapse, and the influencing factors are presented in detail. Furthermore, the implementation of the electrolyte-gated organic synaptic transistors in the emulation of biological perception systems such as olfaction, vision, hearing, taste, and touch, as well as the spiking dynamics of neurons is introduced (Fig. 1). Finally, we discuss the current challenges and possible strategies for constructing reliable, functionalized artificial neuromorphic devices.

## Electrolyte-Gated Organic Synaptic Transistors (EGOST)

**Fig. 2:**
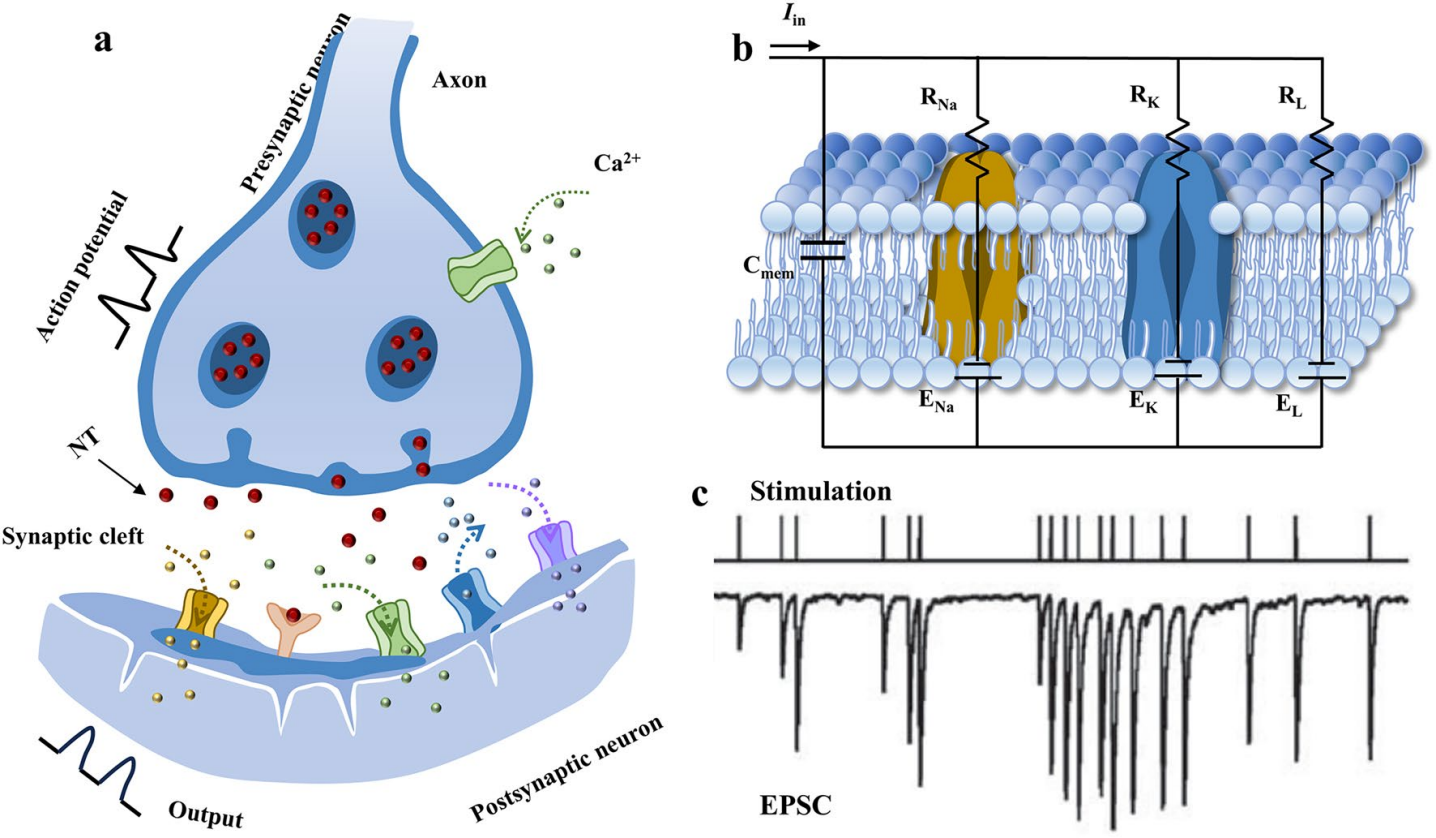
Information transmission for biological neurons. **a** Schematic of a biological synapse including the release of neurotransmitters and ionic fluxes toward the postsynaptic terminal. **b** Electrically equivalent circuit of the cellular membrane based on the Hodgkin–Huxley model. **c** Response of excitatory postsynaptic potential to the stimulus of spike train recorded from the basal ganglia of an awake. Reproduced with permission [44]. Copyright 2000, Society for Neuroscience

Synapse, as a unique and critical biological structure in the human brain, bridging the axon of one neuron (called the presynaptic neuron) and the dendrite of the other one (called the postsynaptic neuron), enables a range of biological activities including the transmission of electrical or chemical information, learning and memory, by modulating ionic fluxes and biomolecules between different neurons. (Fig. 2a) [42]. The electrical synapses in the nervous system could transport nerve impulses directly between the presynaptic and postsynaptic membranes by rapid electrical coupling, corresponding to the transmission, encoding, and filtering of the neural signal. In contrast, chemical synapses which use neurotransmitters to transport signals are much slower but can precisely control the synaptic strength, accounting for the learning and memory behavior [43].

The cell membrane of presynaptic and postsynaptic neurons can be described by the Hodgkin–Huxley model (Fig. 2b), where the ion/biomolecule transmission across the membrane is realized through voltage-gated channels. When a positive electrical stimulus is applied to the presynaptic neurons, the voltage-gated calcium (Ca^2+^) channel opens, allowing the influx of Ca^2+^, which triggers the release of neurotransmitters from vesicles accumulated at the presynaptic terminal. These neurotransmitters dock to the receptors on the postsynaptic membrane, altering the permeability of sodium (Na^+^) and forming a local ionic current and action potential via the influx and efflux of ions across the cell membrane to modulate the conductance of the postsynaptic terminal. Changes in the strength of the communication between presynaptic and postsynaptic neurons based on the history of synapse activity are known as synaptic plasticity, and changes in the conductance of the postsynaptic terminal are regarded as synaptic weight (Fig. 2c) [44].

To emulate the dynamics of neurons, ideal neuromorphic devices should be able to recognize the chemical/ionic signals from electrophysiological fluids and adjust their conductance accordingly to exhibit the plasticity and non-volatility described in biological neurons. However, the hardware implementation of the state-of-the-art neuromorphic device based on two-terminal memristors in neural networks suffers from high write noise and operation voltage, while three-terminal metal–oxide–semiconductor transistors are limited by the complicated wiring in the electrical circuit, resulting in high power dissipation. In order to achieve this goal, it is crucial to develop innovative neuromorphic devices that exhibit low power consumption, operate through mechanisms akin to those of biological systems, and are adapted to environments similar to their natural counterparts.

### Architecture of Neuromorphic Devices Based on Electrolyte-Gated Organic Transistors

**Fig. 3:**
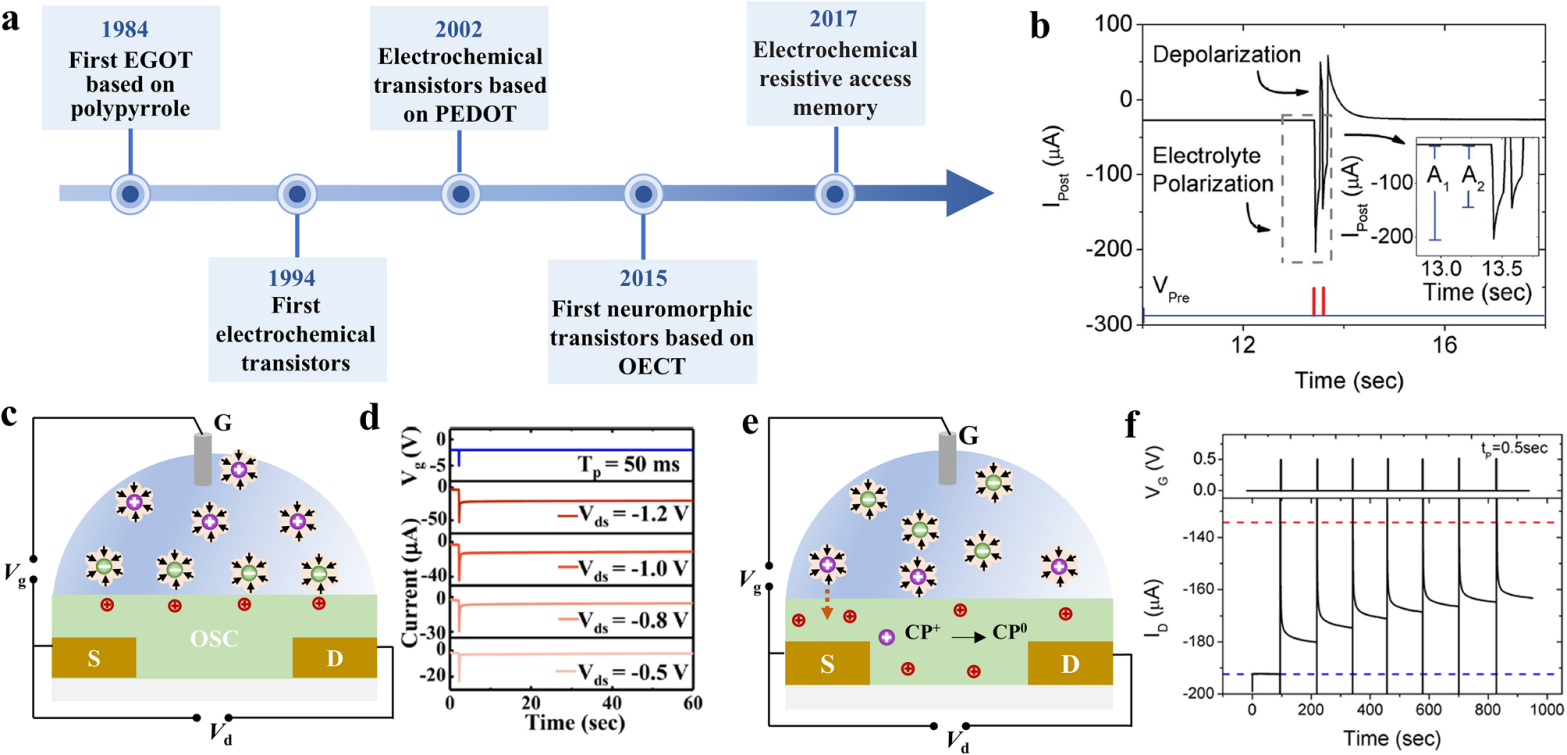
Electrolyte-gated organic transistors for artificial synapse. **a** History of electrolyte-gated organic transistors. **b** Response of postsynaptic drain current to a pair of presynaptic pulse. Reproduced with permission [32]. Copyright 2015, Wiley–VCH. **c** Schematic diagram of organic thin-film transistors in the electrostatic mode and **d** dependence of the postsynaptic current to the applied drain voltage. Reproduced with permission [50]. Copyright 2018, American Chemical Society. **e** Schematic diagram of organic electrochemical transistors in electrochemical mode and **f** response of postsynaptic current to a train of stimulated pulse. Reproduced with permission [36]. Copyright 2015, AIP Publishing

EGOTs represent a significant advancement in organic electronics, combining the properties of organic materials with electrolyte gating mechanisms, which proved to be versatile elements for the applications in electrophysiology, ion detection, and neuromorphic computing. The concept of EGOTs was first proposed by Wrighton et al. in 1984 using the reversible electrochemical switching of electronic conductivity in polypyrrole (Fig. 3a) [45]. Since then, other polymers, such as poly(3-methylthiophene) [46] and polyaniline [47], were applied as the active materials in electrochemical transistors. In 1994, the first electrochemical transistor was partially manufactured using printing techniques, which was then utilized as a microelectrochemical enzyme transistor for glucose and peroxide sensing [48, 49]. In the early 1990s, PEDOT was explored for its potential as an electronic ink and coating material, showcasing a wide range of applications. When doped with PSS, the conducting polymer exhibited high conductivity and redox stability. In 2002, organic transistors based on PEDOT was first reported, showing high current on/off ratio and transconductance, as well as low operation voltages [31]. Continued development of new organic materials with improved electrical, mechanical, and electrochemical properties has broadened the applications of EGOTs as chemical and biological sensors due to their high sensitivity and rapid response. The first neuromorphic device based on PEDOT:PSS OECT was reported in 2015 to mimicking basic synaptic functions, such as paired-pulse depression, adaption, and dynamic filtering [32]. An attractive architecture for organic neuromorphic device combines a battery and OECT was demonstrated in 2017, enabling low power consumption and nonvolatile features [33].

In view of the structural nature, EGOT-based neuromorphic devices feature physically separated input and output terminals, which mimic the synaptic structure of biological neurons. The EGOT can be constructed with different device architectures depending on the position of gate electrode relative to the organic channel, including top-gated, bottom-gated, side-gated, and floating-gate EGTs. The detailed description of each architecture and their corresponding applications could refer to the literature [51]. The operating voltage of EGOT is low, ranging from a few volts to less than 1 V, due to strong electrostatic interactions between the ions in the electrolyte and the gate/organic channel materials, which is crucial for achieving a low-power neuromorphic device. Another fascinating feature is the ease of fabrication process compared to conventional thin-film field-effect transistors. The gate electrode of EGTs could be flexibly positioned either in front of the organic channel or coplanar with the source/drain channel since the charge modulation is governed by the accumulation or depletion of ions in the electrolyte [52, 53]. Furthermore, given that information processing in the brain takes place within a shared electrochemical environment, and that the overall functionality of neuronal networks is governed by global environmental parameters such as ion and neurotransmitter concentrations, homeoplasticity plays a pivotal role in determining the collective behavior of large neural ensembles. However, the connection of solid-state neuromorphic devices is realized by a predefined physical wire network, which severely limits the number of interconnections and results in bulky circuits. In contrast, electrolytes could provide effective interconnections between individual devices and facilely realize global regulation of the synaptic behaviors without complicated wire interconnection, thus mimicking the homeoplasticity of the biological neurons [54].

The organic semiconductors in EGOT are highly attractive replacements for conventional Si-based devices due to its biocompatibility and tunable electrical and mechanical properties which can be achieved through facile chemical synthesis. Furthermore, their flexibility allows for direct interfacing with biology, opening up new possibilities in neuromorphic sensing, actuation, and closed-loop control in bioelectronics. The application of pulse on the top gate electrode causes the injection of cations from the electrolyte into the PEDOT:PSS layer, resulting in the changes in channel conductance. After removing the pulse, the injected cations return to the electrolyte, analogous to the depolarization of cell membrane (Fig. 3b) [32]. According to the ionic permeability of organic channel materials, the EGT can be divided into two categories.

Ion-impermeable organic semiconductors allow for ionic–electronic interaction solely at the interface. Once the gate bias is applied to the electrolytic gate insulator layer, the charged ions accumulated at the electrolyte/organic channel interface. This induces the movement of opposite polarity charge carriers toward the interface, forming the electric double layer (EDL), which then modulates the carrier concentrations and thus alter the conductance of the organic channel (Fig. 3c). These devices are termed as organic field-effect transistors (OFETs) and operate in electrostatic mode. Once the gate bias is removed, the ions at the interfaces immediately diffuse back to the electrolyte driven by the concentration gradient, restoring the channel's original conductance as shown in Fig. 3c. Fu and the colleagues demonstrated in ion gel-gated P3HT OFET, nonvolatile effect occurred only when the presynaptic spike was over − 2 V and the current response of the postsynaptic terminal depended on the applied *V*_ds_. With increasing the *V*_ds_, more carriers were injected into the channel, resulting in enhanced synaptic weight modulation (Fig. 3d) [50]. In addition, the EDL effect of OFET device mainly depends on the dielectric constant of the electrolyte. Kim and the colleagues employed HfO_2_ as the dielectric layer, which had higher dielectric constant than typically utilized SiO_2_, thus a strong EDL was readily generated with lower power consumption, around 0.26 pJ [55]. Table 1 lists the most commonly used organic materials for electrolyte-gated transistors. These include pentacene and organic semiconductors, such as poly(3-hexylthiophene) (P3HT), as well as poly(N-alkyl diketopyrrolopyrrole dithienylthieno [3,2-b]thiophene) (DPP-DTT).

**Table 1:** Organic materials utilized in electrolyte-gated transistors

		Materials	Capacitance (F cm^−2^)/(F cm^−3^)	Electrolyte	*I*_on_/*I*_off_	*τ*_on_	References
Ion-impermeable OSC		P3HT	10 ~ 100 × 10^–6^	polystyrene-b-poly(ethyl acrylate)-b-polystyrene (SEAS) an ionic liquid, 1-ethyl-3-methylimidazolium bis(trifluoromethylsulfonyl)imide ([EMI][TFSI])	10^6^	–	[56]
		DPP-DTT	7 × 10^–6^	100 mM PBS aqueous solution	2400	–	[57]
		Pentacene	7.8 × 10^–6^	0.1 M NaCl aqueous solution	300	4.6 ms	[58]
Ion-permeable OSC	Conjugated polymers (p-type)	PEDOT:PSS	39 ± 3	0.1 M NaCl aqueous solution	10^5^	100 µs	[59]
		PEDOT:TOS	136 ± 50	0.1 M KCl aqueous solution	4.5 × 10^4^	170 ms	[60]
		PEDOT:PSTFSILi100	26 ± 10	0.1 M NaCl aqueous solution	–	90 ± 25 µs	[61]
		PEDOT:PMATFSILi80	27 ± 7	0.1 M NaCl aqueous solution	–	178 ± 3 µs	
		P(g2T-TT)	241 ± 94	0.1 M NaCl aqueous solution	10^5^	420 μs	[62]
		P(gBDT-g2T)	77 ± 23	0.1 M NaCl aqueous solution	10^4^	–	[63]
	Conjugated polymers (n-type)	PBFDO	755	0.1 M NaCl aqueous solution	10^3^	1.8 ms	[64, 65]
		BBL152	588.63	0.1 M NaCl aqueous solution	4.4 × 10^5^	0.38 ms	[66]
		p(gNDI-gT2)	397	0.1 M NaCl aqueous solution	3.21 × 10^3^	5 ms	[67]
		f-BTI2TEG-FT	443	0.1 M NaCl aqueous solution	1.3 × 10^3^	272 ms	[68]
		P(NDIMTEG-T)	250.9	0.1 M NaCl aqueous solution	–	–	[69]
		P(gTDPP2FT)	156 ± 24	0.1 M NaCl aqueous solution	5 × 10^6^	1.75 ms	[70]
		f-BTI2g-TVTCN	170 ± 22	0.1 M NaCl aqueous solution	10^5^	33 ms	[71]
	Conjugated Polyelectrolytes	PEDOT-S	–	BMIMBF4	1.5 × 10^3^	422 ± 15	[72]
		PCPDTBT-SO_3_K	1.34 × 10^2^ F cm^3^	0.1 M KCl aqueous solution 6 M NaCl aqueous solution	6.76 × 10^3^	3.8 ms 4.8 s	[71] [73]
		PEDOT-S:(Oct)_2_NH_2_	–	1 M KCl aqueous solution BMIM:BF4	(7.2 ± 6.5) × 10^2^ (1.3 ± 5.0) × 10^2^	87 ± 59 ms 574 ± 348 ms	[21]
		PTHS	124 ± 38	0.1 M NaCl aqueous solution	–	0.4 ms	[74]
		PTEBS		0.1 M NaCl aqueous solution	(3.1 ± 1.7) × 10^2^	6900 ms	[72]

P3HT, poly(3-hexylthiophene); DPP-DTT, poly(N-alkyl diketopyrrolopyrrole dithienylthieno [3,2-b]thiophene); PEDOT:TOS: poly(3,4-ethylenedioxythiophene) doped with iron(III) *p*-toluenesulfonate; P(g2T-TT): poly(2-(3,3′-bis(2-(2-(2-methoxyethoxy)ethoxy)ethoxy)-[2,2'-bithiophen]-5-yl)thieno[3,2-b]thiophene); PBFDO, poly(benzodifurandione); BBL, poly(benzimidazobenzophenanthroline); PTHS: poly(6[thiophene-3-yl]hexane-1-sulfonate) tetrabutylammonium

In the case of ion-permeable organic semiconductors, ionic–electronic interactions take place in the entire bulk of the organic channel. This is because the ions can penetrate into the organic film and modulate the conductance of the whole bulk via electrochemical doping/de-doping, generating three-dimensional volumetric capacitance. The corresponding device is classified as organic electrochemical transistors (OECTs) (Fig. 3e). Unlike electrolyte-gated organic field-effect transistors with their volatile channel conductance at low presynaptic voltage, OECTs require reversed gate voltages and long time to extract the penetrated ions from the channel and restore its original conductance, as shown in Fig. 3e. Thus, the conductance states of the OECT device under stimulation are strongly correlated with the doping states of the organic channel, which is a critical building block for neuromorphic computing. Gkoupidenis and the colleagues demonstrated the implementation of neuromorphic functions similar to biological memory using OECT with a poly(tetrahydrofuran)-based PEDOT derivative (PEDOT:PTHF) (Fig. 3f). Different from the alteration of doping level in PEDOT:PSS, a conformational change of the polymer structure occurred in PEDOT:PTHF upon applying a high reduction potential (0.6 V). The successive application of gate pulse resulted in the device response exhibited relaxation to multiple states of memory, and the device exhibited a gradual transition from short-term to long-term memory when the pulse number was over 3 [36]. The most commonly used channel materials for OECTs are conducting polymers such as poly (3,4-ethylenedioxythiophene)-poly (styrenesulfonate) (PEDOT:PSS), poly(2-(3,3′-bis(2-(2-(2-methoxyethoxy)-ethoxy)ethoxy)ethoxy)-[2,2′-bithiophen]-5-yl)thieno[3,2-b] thiophene (Pg2T-TT), poly(benzimidazobenzophenanthroline) (BBL), and poly (benzodifurandione) (PBFDO).

To improve the solution processability of organic semiconductors, long alkyl or glycolated side chains are normally introduced. However, this approach is not only detrimental to capacitance, but also disrupts the pristine conformational structure of the semiconductor due to a significant swelling of the organic film, originating from the influx of hydrated ions and incorporation of charge traps when contacting physiological fluids [64, 75]. Therefore, the organic semiconductors with shorter side chains or without side chains are the preferred option to achieve superior device performances. A broad overview on EGOTs and their applications in bioelectronics is provided by a previous review [51].

The gate electrode and organic semiconductor channel can be regarded as the presynapse and post-synapse, respectively, when considered in conjunction with the structure of a biological synapse. Applying a gate bias to the gate electrode causes ions in the electrolyte to migrate, regulating the channel conductance and effectively adjusting synaptic weights. Changes in synaptic weights depend on the history of synapse activity, referred to as synaptic plasticity. This is closely linked with the foundation of learning and memory of neuronal systems. The electrolyte-gated synaptic transistors are the promising candidates for neuromorphic applications. They enable facile signal integration, with synaptic plasticity pre-tunable during fabrication rather than dynamically adjusted during operation. This is in contrast to the traditional synapse circuits composed of several transistors and a capacitor, which perform each input separately.

### Neuromorphic Devices to Handle Neurochemical Signals

In biological neuron networks, neurochemicals such as neurotransmitters or ions are indisputably critical for information encoding and conveying. A neurochemical-mediated artificial neuron comprises three essential components: a recognition functionality sensitive to the chemical input (the function of a biosensor), a memristor, and a warehouse storing the neurochemical. It can be integrated with other devices, such as biomedical care systems, and transmit signals into and receive stimuli from artificial or biological systems, including the brain. Neurochemical-mediated artificial neurons selectively respond to specific chemical inputs, unlike physical stimuli neuromorphic chips. The interaction of the neurochemical with the stimuli-sensitive material alters its properties, including conformation switching, capacitance changes, and optical property changes. These are then converted into current modulations. It is important to note that in some cases, neuromorphic material can perform all three functions: stimulus sensitivity, transduction, and memorizing. The memristor is a crucial component of the neuromorphic device. It determines the plasticity, the memorization time (long or short term), and the type of neuromorphic activity (inhibitory or excitatory). When the current from the transducer exceeds a memristor-dependent threshold, the memristor transmits a signal into the warehouse. The neurotransmitters stored in the warehouse are released into the input-sensitive environment.

It is important to note that the conversion of chemical inputs into neuromorphic information is crucial not only for interfacing nervous tissue but also for the digitalization of the senses in general. This refers to the process of converting sensory inputs into digital data, which can then be utilized by digital systems. The sheer number of sensory inputs presents a significant challenge to traditional signal processing in terms of efficiency and power consumption [76]. Chemical-mediated neuromorphic devices will become a powerful tool in multi-parameter sense digitalization.

Various strategies have been reported to emulate neurochemical signals in neuromorphic computing, including ion- or neurotransmitter-dependent conductance modulations in memristors. Development of neuromorphic devices that translate chemical signals into history-dependent conductance modulation by varying the chemical and structural composition of the neuromorphic material will facilitate plasticity, memory, learning, and sense digitalization. These neuromorphic materials must be capable of mimicking the propagation of information in neural networks. The transmission of action potentials over the synaptic junction in chemical synapses is controlled by the release of neurotransmitters, which is triggered by the variation of ion concentrations such as Na^+^, K^+^, Cl^−^, Ca^2+^, and H^+^ near the synaptic cleft.

#### Proton-Modulated Neuromorphic Devices

It is a well-established fact that H^+^ affects ion channels and neurotransmitter receptors [77], modulating action potential transmission as a result. The importance of protons as regulators of neural information processing inspired the implementation of proton-dependent neuromorphic devices. Transition metal oxides (TMO), including WO_x_, VO_x_, and NiO_x_, constitute a prominent class of materials that show proton-dependent variations of conductivity. These TMOs undergo a H^+^-dependent transition, changing from a conductor to an insulator. Annealing, electrochemical doping, and electrolytic gating are the methods to use to initiate this transition. A proton-hopping process is the mechanism by which protons are transported in the material, involving the oxygen atoms and vacancies [78]. While the material conductivity can change by several orders of magnitude, it is not compatible with aqueous media. Proton-conducting 2D materials like graphene implemented in a field-effect transistor work differently. This graphene field-effect transistor employs the transport of hydrogen ions across the graphene layer to the CaF_2_ interface beneath it [79]. The interface was charged, which facilitated spike-time-dependent plasticity. In 2D α-phase MoO_3_, electrochemical doping was used to successfully emulate depression and potentiation of synaptic weight, as well as the transition of short-term plasticity to long-term potentiation via the history of an electric field-driven electrolysis of water present in an ionic liquid [80].

A third class of proton-conducting neuromorphic materials are synthetic organic materials featuring biocompatibility and flexibility, which are the best choice for interface BNNs among the proton-conducting materials. Hydrogen-bonded organic frameworks (HOFs) are a subgroup of proton-conducting organic neuromorphic materials that form crystalline structures based on non-covalent interactions. For some HOFs, it was proved that the resistive switching was directly ascribed to an interaction of water with hydrogen bonds formed in the organic crystal [81]. Hydrogel-based electrochemical transistors have also been shown to undergo conductance modulation via hydrogen bond reconfiguration, where the hydrogel acts as a proton-conducting electrolyte and is in contact with the PEDOT:PSS channel layer. Furthermore, the hydrogel can be substituted by other proton-conducting materials, such as polymers. In one example, Nafion was used as a proton-conducting electrolyte to enable a potential-driven protonation of a poly(ethylenimine) film and a subsequent reduction of a neighboring PEDOT:PSS channel, which induced a decrease in the channel conductance [33]. Proton-conducting organic materials facilitate low switching energies, large switching speeds, and a large number of write–read cycles [82].

#### Ions Modulate Neuromorphic Devices

Ion-modulated neuromorphic devices deliver electronic signals by coupling the motion of ions under a gating effect [51]. Under a gating potential, ions move toward electrodes and change their charging state, which is the writing step. The "read" operation is straightforward: Ions are held and electrode charges are maintained when removing the potential, since they are blocked by an ion-conducting/electron-blocking electrolyte [83].

OECTs are the ideal choice for ion-modulated organic electrochemical neurons (OECNs) thanks to their low operation bias, high transconductance, and biocompatibility [32]. To achieve a superior ion–electron coupling and ensure the retention of the induced state, the gate electrode, typically composed of a metal presynaptic material, is coated with OMIEC or ionic polymers. A postsynaptic behavior can be simulated by a continuous and variable conductance of the channel, which is manipulated by a tunable gate potential. A small bias on the gate electrode forms an electrical double layer between the channel surface and the electrolyte, causing ions to rapidly drift back after switching the potential. This accounts for short-term plasticity. Increasing the gate voltages achieves long-term plasticity. This allows ions to inject into and couple with OMIEC, resulting in a quasi-permanent change of conductance [33, 84]. Furthermore, the specific characteristics of different OMIEC molecules are used to improve the performance of OECNs. This includes spike-timing-dependent plasticity, spike-rate-dependent plasticity, and short-/long-term potentiation.

Gkoupidenis and his colleagues have demonstrated that an OECN based on PEDOT:PSS can be used to implement depressive short-term synaptic plasticity functions [32]. A depletion-mode OECT is always in the on-state. The current drop is caused by the injection of cations, which compensate for the PSS group and induce a chemical reduction of the PEDOT^+^ backbone. This property causes high operating currents (in the low mA range) and a high operating gate voltage (*V*_G_ approximately + 0.8 V vs Ag/AgCl), which switch off the device and increase the power assumption [85, 86]. However, a high *V*_G_ also results in instability for devices in an aqueous electrolyte, often leading to parasitic reactions with water and oxygen and ultimately device degradation. PEDOT:PSS can be de-doped by organic molecules with amino functional groups. The use of treated PEDOT:PSS as channel material results in a significant threshold voltage drift toward low gate voltage. Furthermore, the nonvolatile ability can be improved due to the low switching voltages.

Burgt et al. used PEI-de-doped PEDOT:PSS as the channel material. At a positive presynaptic potential (writing step), cations were driven into the postsynaptic region and protonated PEI simultaneously. Electrons flowed through the external circuit and removed holes in the PEDOT^+^ backbone. The subsequent reduction in channel conductance was clear. This reaction can be reversed by applying a negative gate voltage. PEI was instrumental in forming and retaining the PEDOT in the postsynaptic electrode in its neutral state, preventing oxidation during the read [33].

It is also noteworthy that OECNs based on poly(benzimidazobenzophenanthroline) (BBL) display a reversible Gaussian-shaped transfer curve under moderate gate bias. Harikesh and colleagues demonstrated that different ion species and concentrations can shift the transfer curve to varying degrees [87]. They successfully fabricated Na^+^ activating OECN (Na-OECT) and K^+^ activating OECN (K-OECT) and implemented them into a circuit. These OECNs demonstrated a switching speed of 0.5–1 ms, which is comparable to the activation of ions in biological neurons. When a current was applied, the Na-OECN reached its maximum conductance and transmitted the current to activate the K-OECN. The higher and longer-lasting current in the K-OECN design meant that the output was taken below the resting value of 175 mV in a short time. The circuit output potential displayed typical features of a biological action potential when a constant current was input.

**Fig. 4:**
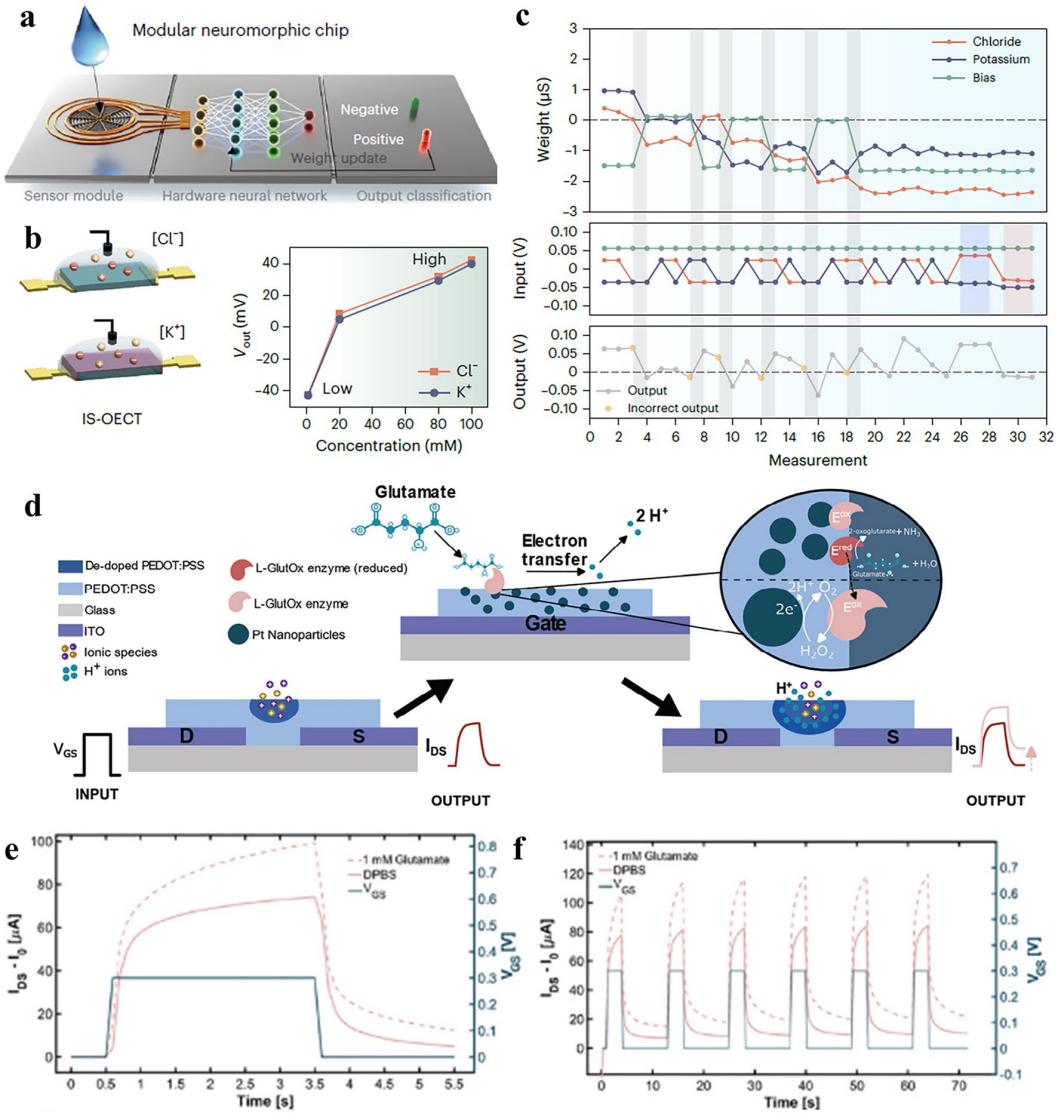
Neuromorphic device to handle chemical signals. **a** Schematic illustration of the modular biosensor with distinct functions. **b** Illustration of ion-selective OECT sensors for measuring K^+^ and Cl^−^ concentrations. **c** Synaptic weight update during a training cycle using ion-selective electrodes. Reproduced with permission [90]. Copyright 2023, Springer Nature. **d** Schematic illustration of post-synapse response of electrochemical neuromorphic device to glutamate, channel current response to **e** a single voltage pulse and **f** sequential voltage pulses. Reproduced with permission [91]. Copyright 2024, Wiley–VCH

Therefore, incorporating OECTs into circuits is a more effective approach than using single OECT alone. It allows us to achieve more complex functionality and move closer to developing technology that is relevant and viable for application goals [88]. Cea and colleagues have demonstrated that the integration of depletion-mode and enhancement-mode OECTs can be combined to create a nonlinear rectification circuit [89]. Doremaele and colleagues developed a modular biosensor consisting of a sensor input layer, a network of neuromorphic devices formed by ion-selective OECTs, and an output layer for classification of K^+^ and Cl^−^ from modified donor sweat (Fig. 4a–c). The output signal from OECT hardware could power light-emitting diodes once reached a threshold activation function and indicated a negative or positive diagnosis of the input samples [90].

Apart from the material optimization, the geometry strongly influences the performance of OECNs. From the OECT formula, it is clear that the channel length is inversely proportional to the conductance, which affects the postsynaptic characteristic. Furthermore, the shaping of an artificial synapse depends on ion mobility. Ions are driven toward the postsynaptic electrode with a potential applied, but the mobility of ions is slow in aqueous, not to mention in solid electrolytes. Therefore, it is necessary to shorten the distance between the pre- and postsynaptic electrodes to improve the time response of OECNs by facilitating faster ion transport.

#### Neurotransmitter-Modulated Neuromorphic Devices

Synaptic conditioning, including biochemical signaling activity, is essential for implementing biologically integrated neuromorphic systems. A variety of materials have been used to create neuromorphic devices that can perform specific tasks of artificial neurons. The most commonly used neuromorphic platforms are based on organic materials. These platforms demonstrate both short-term and long-term synaptic behaviors derived from the redox reaction of electroactive neurotransmitters such as dopamine [92]. The redox-active neurotransmitters added externally react with the organic channel material, altering its conductance properties in a dose-dependent manner. Keene et al. took the next step in brain-inspired neuromorphic systems, directly coupling a neuromorphic device made from an OECT with dopaminergic PC-12 cells [4]. The goal was to create a biohybrid synapse that demonstrates cell-dependent neurotransmitter-mediated synaptic plasticity, with the neurotransmitter signal originating from cells, paving the way for combining artificial neuromorphic systems with biological neural networks. In another application, a similar OECT platform was used to modulate artificial synapses using dopamine signals. This device had an integrated closed-loop system and was able to control a robotic hand using adaptive and reinforcement learning [93].

Two major classes of materials in neuromorphic devices, namely nanoparticles and organic films, have recently been merged to enable interaction with non-electroactive species present in the central nervous system, such as glutamate signals, as shown in Fig. 4d-f [91]. A conductive PEDOT:PSS film was therefore functionalized with an enzyme (glutamate oxidase) and platinum nanoparticles. The latter catalyzes the oxidation of H_2_O_2_ signals originating from neurotransmitter-specific enzymatic reactions, enabling neuromorphic functions driven by non-electroactive neurotransmitters. The enzyme involved not only facilitates the chemical conversion from a non-electroactive into an electroactive agent, but also provides high selectivity over other neurochemicals.

This is a crucial aspect, as biological neural information processing typically involves multiple neuromodulators. Various organic neuromorphic devices have been developed that are capable of recording two separate neurotransmitter signals via specific interfacial electrochemical reactions with minimal cross talk. One example is an electrochemical platform that effectively transduces excitatory dopamine and serotonin signals into reversible and non-reversible variations of PEDOT:PSS conductance [94]. Kim et al. demonstrated that an artificial synapse can be used to control the conductance of a PEDOT:PSS channel by applying an excitatory stimulus, acetylcholine, which increases the channel conductance, and an inhibitory signal, adrenaline, which reduces it [95]. Various synaptic functions including EPSC, IPSC, PPF, LTP, LTD, and STDP were emulated by balancing the level of the excitatory and inhibitory neurotransmitter signals in combination with electrochemical gating. An advanced neurotransmitter co-modulation was achieved by optimizing the biorecognition process via the assembly of a lipid bilayer on an electrochemical neuromorphic organic device to resemble postsynaptic structural and functional features of living synapses. Synaptic conditioning was established by dopamine and signals to irreversibly change the device conductance, thus achieving Pavlovian associative learning [96].

The characteristics of the neuromorphic device depend on two factors: the redox properties of the neurotransmitter and the chemical nature of the organic material. In addition to neuromorphic systems made from PEDOT:PSS, a number of other organic materials have been developed, including a polymer membrane comprised of poly(diallyl-dimethylammonium chloride) and poly(3-sulfopropyl acrylate potassium salt). This mixed polymer can be controlled by the exposure to acetylcholine to control its ionic permeability. The neutralization of charges in the membrane by the zwitterionic ACh is concentration-dependent and modulates the electrical device characteristics, emulating the signal transmission behavior of biological neurons [97].

In addition, small molecule OMIECs have been successfully demonstrated using a linear oligoether as an n-type acceptor molecule with optimized butyl-side chains. These devices successfully modulate synaptic behavior and accurately sense excitatory dopamine and inhibitory glycine signals [98]. The synthetic tailoring of the organic material also allows for the combination of different stimulus modalities. A dual-gate organic synaptic transistor with a photoconductive polymeric semiconductor, a ferroelectric insulator of P(VDF-TrFE), and an extended-gate electrode functionalized with boronic acid was used to successfully detect the neurotransmitter dopamine and polychromatic light, enabling stimulus-induced memory consolidative artificial synapses [99].

Recently, conductance-based organic electrochemical neurons were demonstrated that can spike at biorealistic frequencies nearing 100 Hz at random [87]. They can also modulate spikes based on neurotransmitters and ions, which can be used to stimulate biological nerves in vivo. These OECT-based devices use a mixed ion–electron conducting ladder-type polymer, poly(benzimidazobenzophenanthroline), which is stable, ion-tunable, and anti-ambipolar. The latter is used to emulate the activation/inactivation of sodium channels and delayed activation of potassium channels of biological neurons with great success.

## Requirements to Achieve High-Performance Artificial Synapses

In biological nervous systems, numerous neural signals are transmitted in a massively parallel, resilient, error-tolerant, and energy-efficient method [41]. The response of postsynaptic neurons to the presynaptic neurons through synapse varies with the external stimulations, such as temperature, pressure, and photoelectric stimulations [43]. The synaptic plasticity is straightforwardly related to the data processing, information transmission, and memory which are all by ion and neurotransmitter signals [100]. To emulate efficiently the parallel operation and interconnectivity of the brain, the desirable artificial synaptic transistors must feature the following metrics: operate with low power consumption, multiple conductance states for on-chip learning, tunable state retention time, and capability to handle chemical signals to satisfy specific requirements and applications.

### Low Power Consumption

It is indisputable that one of the most significant metrics of bioinspired organic artificial synapses exceeding the limitations of the von Neumann computer architecture is their remarkable ability to consume minimal power [2]. A biological nervous system is comprised of electrical and chemical synapses [43]. The electric synapses generate action potential by the consumption of 10^9^ ATP molecules (100 pJ) per action potential [101]. In a chemical synapse, the neurotransmitters diffuse from the presynaptic membrane to the postsynaptic membrane through the synaptic cleft (20–40 nm), which is captured by the membrane and stimulates the opening of ion channels. This process consumes around 10 fJ per synaptic event.

The operation of an artificial synapse can be divided into two steps: programming (i.e., the inputs from the presynaptic neuron: writing or switching) and reading (the response of postsynaptic neurons). The energy consumption (*E*) for a synaptic synapse is the product of voltage and the integral of corresponding current with the duration of programming pulse *t* [2]. For the calculation of *E* of three-terminal-based organic synaptic transistor, both gate voltage/current (*V*_g_/*I*_g_) and drain voltage/current (*V*_d_/*I*_d_) should be taken into consideration, i.e., *E* = (*V*_g_ × *I*_g_ + *V*_d_ × *I*_d_) × *t*. Since the operation voltage and the resulting current are recognized to directly depend on the device structure and channel material, the energy consumptions of an artificial organic synaptic device is governed by the device’s architecture/geometry, the conductance of organic semiconductors, and its operation mode. These factors are discussed in the following sections.

#### Influence of Device Architecture on Energy Consumption

**Fig. 5:**
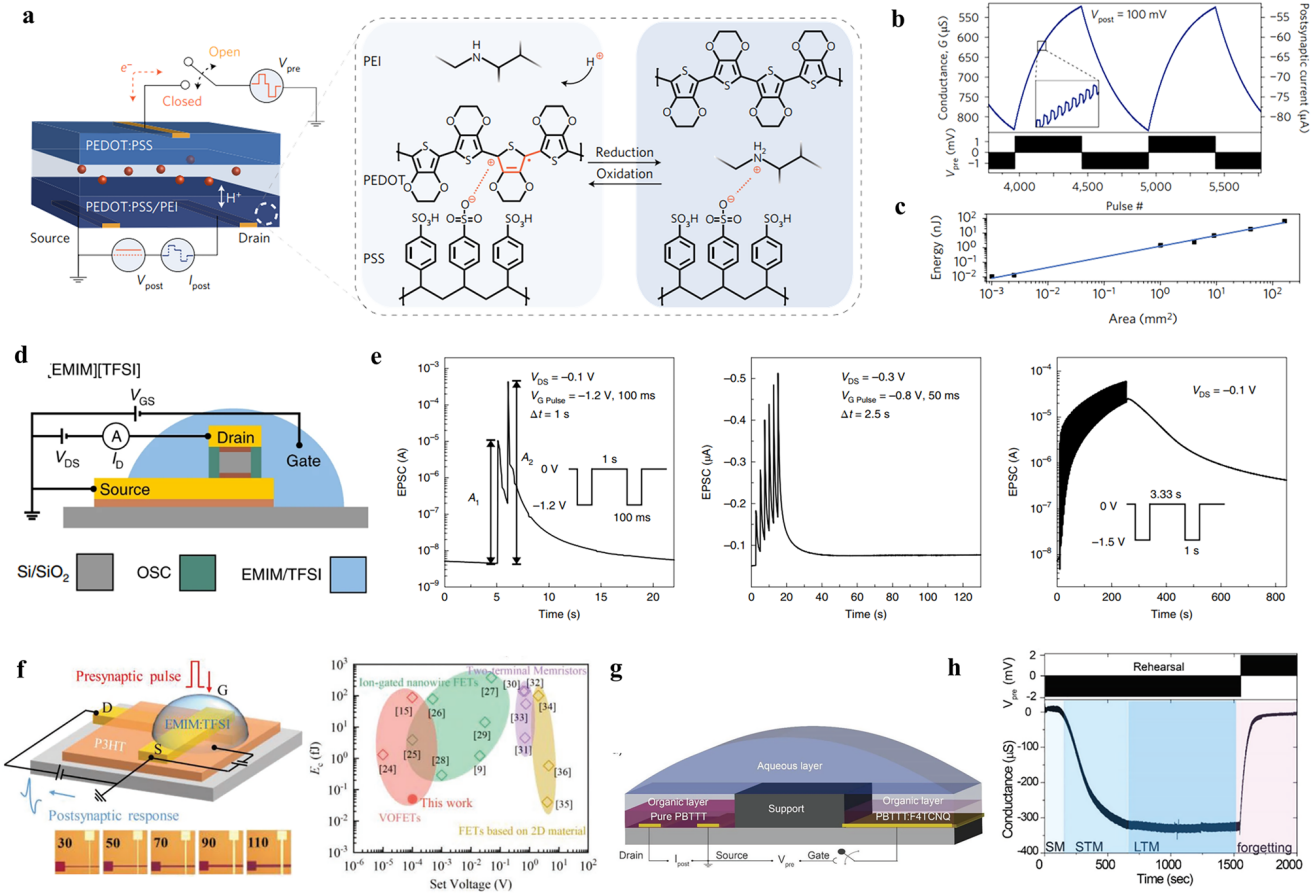
Electrolyte-gated organic synaptic transistors with low power consumption. **a** Sketch of organic electrochemical resistive access memories (OEC-RAMs) structure and the redox reaction of postsynaptic terminal. **b** Long-term potentiation and depression behaviors of the OEC-RAMs. The inset is a zoom-in showing the individual states. **c** Switching energy measured as a function of device area. Reproduced with permission [33]. Copyright 2017, Springer Nature. **d** Device architecture of electrolyte-gated vertical organic field-effect transistors and **e** the emulated synaptic functions. Reproduced with permission [34]. Copyright 2019, Springer Nature. **f** Left: Schematic illustration of vertical organic transistors with electrode widths ranging from 30 to 110 μm. Right: Comparison of various vertical and planar transistors in terms of the energy consumption. Reproduced with permission [39]. Copyright 2022, Wiley–VCH. **g** Schematic of a neuromorphic transistor. **h** Short-term potentiation and depression displaying 250 discrete states. Reproduced with permission [105]. Copyright 2018, Wiley–VCH

Optimization of size and geometry is a flexible and straightforward strategy for tuning the response speed and operating voltage of three-terminal transistors. For the electrolyte-gated organic transistor, the channel conductance is generally proportional to the channel geometry (channel width *w*, length *l*, and film thickness *d*), so *E* can be reduced via minimizing the device size [33]. An *E* with the value of around 10 pJ could be achieved for a battery-like electrochemical transistor with the channel area of 10^–3^ mm^2^, which is expected to be reduced to 35 aJ by downscaling the channel area to 10^–8^ mm^2^ (Fig. 5a–c) [33]. In addition, tuning the channel width/length ratio is able to achieve the maximum transconductance at zero gate bias in planar OECTs or interdigitated OECTs [102, 103], in which case the *E* depends mainly on the drain voltage/current. A vertical architecture is considered a promising structure to reduce the channel dimension and foster device’s miniaturization. Weitz et al. designed a nanoscopic device based on a vertical transistor architecture with diketopyrrolopyrrole–terthiophene donor–acceptor polymer (PDPP) as the prototype semiconductor and ionic liquid 1-ethyl-3-methylimidazolium bis(trifluoromethylsulfonyl)imide ([EMIM] [TFSI]) as the electrolyte. The nanoscopic structure allows the device to operate as a memristive device with an energy consumption below 100 fJ (Fig. 5d, e) [34, 104]. A similar structure was used in the work of Liu et al. with the same ionic liquid as the electrolyte and P3HT cross-linked by 1,6-bis(trichlorosilyl)hexane as the active material. According to the calculation equation of *E*, the spike width (stimulation time) is also a key factor for *E*. By reducing the electrode width from 110 to 30 μm and scaling down the channel length to 30 nm, accompanied by setting low stimulation time (0.1 ms) and driving voltage (0.1 mV), an ultra-low power consumption of 0.06 fJ could be achieved, which is lower than that of biological synapse (1–10 fJ) (Fig. 5f) [39].

Although short pulse widths lead to lower *E*, the stimulation applied should be matched to the desired applications. Fast switching in microseconds or even nanoseconds is not necessary for biological neurons where events occur on the millisecond range, but is required for some sensor applications, especially for the detection of hazards [19, 51]. It is noteworthy that scaling down the device size is usually accompanied by high operating noise and poor device reproducibility, as patterning of organic semiconductors is the main challenge during miniaturization, which is sensitive to conventional photoresists. Therefore, advanced materials and manufacturing techniques, such as in situ polymerization of active materials, utilization of orthogonal photoresists and photopatterning, or inkjet printing, are required to achieve high device resolution/reproducibility and low energy consumption [106].

#### Influence of Organic Channel Materials on E

Organic electronic materials are a promising alternative to conventional inorganic semiconductors for certain neuromorphic applications, since their structure and properties, similar to those of biological systems, could be easily tailored by chemical synthesis, and they could be processed by low-cost solution printing or photolithography fabrication. In an electrolyte-gated organic synaptic transistor, the output current level under the applied driving voltage varies greatly depending on the intrinsic conductivity of the organic channel materials, thus affecting the energy consumption. The p-type polymer polyethylenedioxythiophene and its dopant poly(styrenesulfonate) (PEDOT:PSS), as commercially available conducting polymer, has been widely utilized as channel materials directly interfacing the electrolyte for modern bioelectronics due to its intrinsic high conductivity. Electrochemical synaptic transistors using this polymer as the active channel material generally exhibit high initial currents. They are operated in depletion mode which requires the application of a high gate voltage (~ 0.8 V vs Ag/AgCl) to drive the device into the off-state by an electrochemical de-doping process [32]. Instead, enhancement-mode devices are favorable they could reduce the initial channel current, thus reducing the power consumption.

A few research have been carried out to reduce PEDOT:PSS, such as de-doping with polyethyleneimine (PEI) [33, 107], utilizing aliphatic amines as de-dopant molecules [85, 108]. For example, Burgt et al. demonstrated a novel concept with the device architecture comprising a PEDOT:PSS as presynaptic electrode, PEDOT:PSS/PEI as postsynaptic electrode, and an electrolyte as the synaptic cleft [33]. Once a positive presynaptic potential is applied to the PEDOT:PSS electrode, the penetration of cations from the presynaptic electrode into the organic channel causes the protonation of PEI and the compensation of PSS^−^, thus resulting in the decrease of the polymer conductivity. As mentioned in the previous chapter, this working mechanism combined with the downscaling of the channel size resulted in a low energy consumption of ~ 10 pJ (Fig. 5a–c).

In addition, semiconducting polymers are preferred as the channel material for electrolyte-gated synaptic transistors to achieve devices with low power consumption compared to conducting polymers. Duong et al. utilized semiconducting polymer poly[2,5-bis(3-tetradecylthiophen2-yl)thieno[3,2-b]thiophene] (PBTTT) as the organic channel materials and PBTTT doped by F4TCNQ (7,7,8,8-tetracyano-2,3,5,6-tetrafluoroquinodimethane) as the gate electrode to fabricate a neuromorphic device that operated in enhancement mode and was more suitable for power reduction than conducting polymers (Fig. 5g, h) [105].

In fact, there are other factors to consider when using low-conductivity semiconductors in synaptic transistors, including the signal-to-noise level of the resulting bioelectronics and the long-term stability and durability during operation, especially in the liquid environment.

#### Influence of Operation Mechanism on E

As aforementioned, two types of synapses exist in the biological networks, chemical and electrical synapses, which could separately transmit the chemical and electrical signals from the presynaptic neurons to the postsynaptic neurons. The comparison of the energy consumption of the two synapses was made in a single cell via three gate approaches, using molybdenum disulfide (MoS_2_) as the channel material. It was found that the electronic mode, established via electron trapping/de-trapping at the semiconducting channel, operated at significantly higher power (~ 13 nJ) than the ionotronic mode (~ 4.8 pJ per event). The latter was based on the ion migration–relaxation kinetics at the ionic liquid/semiconductor interfaces, when stimulated with the same presynaptic spike [109].

In the artificial synapse, most of the neuromorphic functions are achieved based on the operating mechanism of corresponding devices including charge trapping [110, 111], electrochemical redox [31, 32], and ion migration [112, 113], which is subdivided according to the architecture and active materials of the devices. Artificial synapses based on the ion migration mechanism are highly desirable because of their directional movement of ions driven by external stimuli, analogous to the ion release process in biological synapses, especially for emulating chemical synapses in an aqueous environment. The electrolyte is usually confined in micro- or nanochannels in fluidic-based systems featuring memresistance and memcapacitance [114]. Xiong et al. developed a polyimidazolium brush (PimB)-confined fluidic memristor by growing the molecules on the inner wall of a pipette. The history-dependent ion memory could be realized by establishing an anions concentration equilibrium and charge balance between the inner and outer PimB upon application of electric or chemical stimulations [112]. The 150-nm-diameter nanopipette-based synaptic device exhibits an energy consumption as low as 0.66 pJ per spike event, which is promising for the application in the biological system. At present, electrolyte-gated organic synaptic transistors based on ion migration are rarely developed, mainly because the ion migration method is limited by the controllable ion migration dynamics [115] and the strong shielding effect of the confined channels in an aqueous solution impedes the interionic interactions, thus limiting the formation of memory functions [116].

Another promising principle for neuromorphic transistors is charge trapping. The charged nanoparticles embedded in the bulk of the organic semiconductor could electrostatically repel the charge carriers in the materials, thereby modulating the channel resistance. As the charge trapping principle provides high channel resistance, it is a promising strategy to achieve synaptic transistors with low power consumption. However, this principle is limited by the trade-off between channel dimension and nanoparticle size, where high transistor density requires a smaller channel and lower the particle concentrations. Furthermore, the nanostructure located at the dielectric–semiconductor interface causes the formation of a disordered semiconductor layer, so an inevitable trade-off between charge transport and memory performance is a predominant issue to be addressed [117].

In electrolyte-gated organic synaptic transistors, electrochemical redox is the prominent sensing mechanism, using the gate electrode to tune the channel conductance via an electrochemical doping/de-doping process. This type of device consists of two circuits. One is the ionic circuit, which describes the ion flow between the gate/electrolyte and electrolyte/channel driven by the gate bias, and the other one is the electronic circuit, which represents the charge carrier transport between the source/drain electrode [118]. A type synaptic transistor relied on this mechanism is organic electrochemical transistors. Gkoupidenis et al. demonstrated the realization of basic neuromorphic functions, including pair-pulsed depression, adaptation, and dynamic filtering, by the PEDOT:PSS-based OECTs with a lateral gating configuration [32, 36]. These short-term synaptic functions are actuated by the dynamics of ion diffusion between electrolyte and the organic channel. Burgt et al. developed an electrochemical neuromorphic organic device with the same configuration as an OECT, using PEDOT:PSS-coated gold as the presynaptic electrode and PEDOT:PSS partially reduced by PEI as the postsynaptic channel material. The application of a positive gate bias drives cations in the electrolyte flow from the PEDOT:PSS presynaptic electrode into the postsynaptic electrode, causing the protonation of PEI and thus the decrease in the conductance for the postsynaptic electrode. As the “read” operation process of the presynaptic electrode was decoupled from the “write” operation via the ionic electrolyte, the battery-like synaptic transistor could operate at extremely low switching voltages, around 10 mV potentiation and depotentiation pulses. Thus, the artificial synaptic device based on the redox mechanism is predicted to switch with only ~ 35 aJ energy when the device is downscaled to submicron sizes [33].

### Accurate Controlling of the Synaptic Functions

Given the structure of biological synapses, the voltage applied to the presynaptic terminal drives the migration of ions to/from the of postsynaptic terminal, thereby altering its overall conductance. The amount of released neurotransmitters and receptors in the postsynaptic membrane concurrently determines the synaptic strength/conductance. Thereinto, the neurotransmitters are at an abnormal level under the circumstances of neurodegenerative diseases or strong emotions. Thus, the realization of linear multi-level conductance states and nonvolatile characteristics are essential to emulate the synaptic behavior and very useful to demonstrate neural network-based pattern recognition and for efficient neuromorphic computing [119, 120]. In electrolyte-gated organic transistors, the conductance of organic channel is a consequence of the applied voltage history and exhibits multiple states that depend on the on-to-off ratio of the source–drain channel [33, 121]. The response of the conductance to the gate pulse depends on the spike voltage, the spike duration, the spike number, and the spike frequency [32, 37, 106].

Synaptic plasticity is a dynamic process that varies from a few milliseconds to months. In terms of timescales, the synaptic functions could be divided into two main categories: short-term plasticity, which represents temporal enhancements of the synaptic weight associated with various computational tasks of brain; and long-term plasticity, which lasts from few minutes to several weeks and governing learning and memory [122]. Short-term plasticity could be converted into long-term plasticity if persistent stimulations are applied to the presynaptic terminal and cause a long-term regulation of the synaptic strength (conductance) of the postsynaptic terminal [36, 123].

Paired-pulse facilitation (PPF), which is a representative short-term plasticity obtained by two consecutive spikes, is essential for decoding temporal information in biological systems. Altering the pulse intervals results in the exponential decay of PPF indices, which is similar to the signaling characteristics of biological systems [32]. Another typical synaptic function associated with learning is the spike-timing-dependent plasticity (STDP), which represents the change in synaptic weight by varying the time difference between presynaptic and postsynaptic spikes [50]. It is the most commonly used function to implement Hebbian learning rules, which are fundamental learning rules for training neural networks for artificial synapses and validating the performance of artificial electronic synaptic devices [121].

**Fig. 6:**
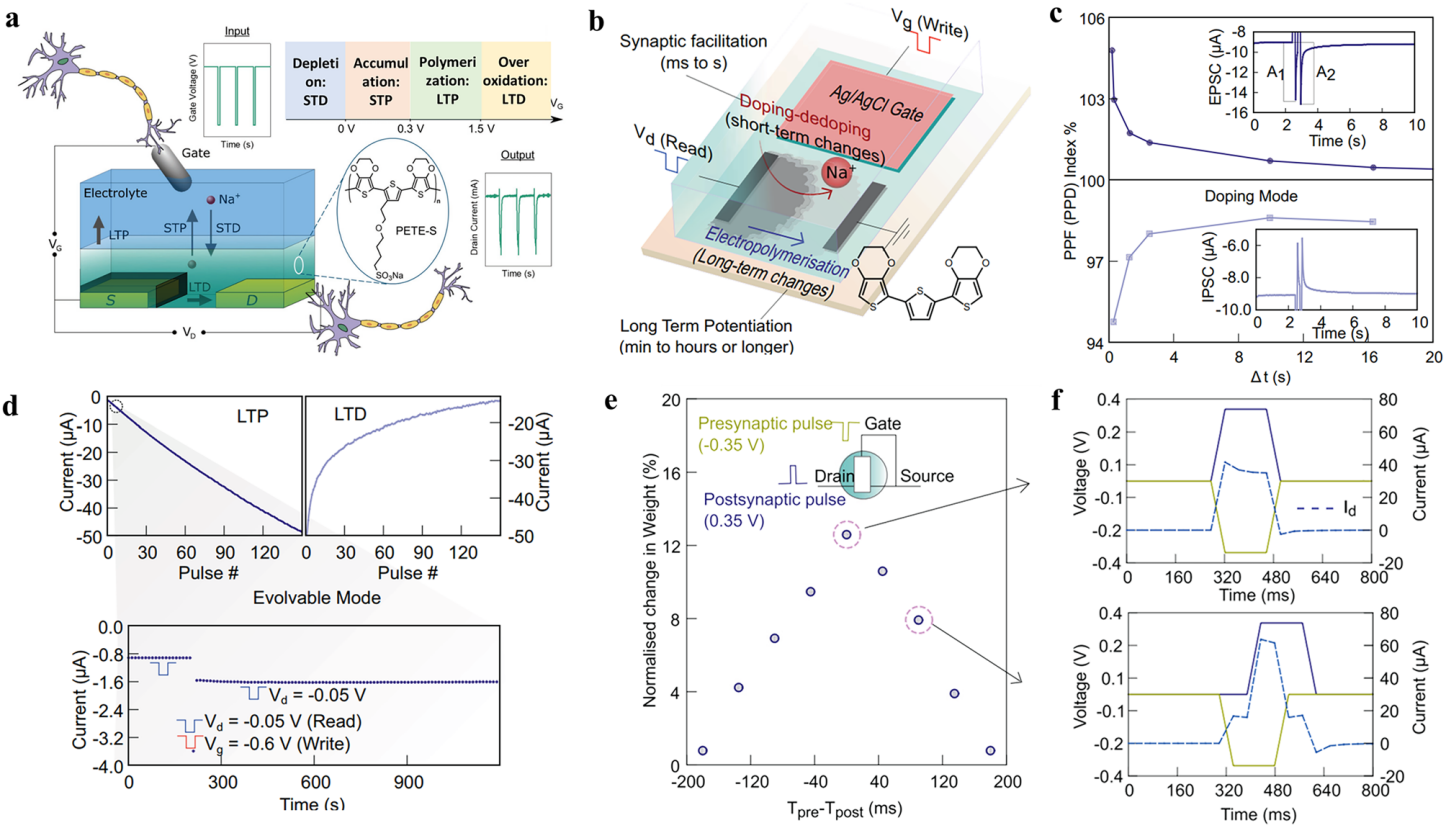
Short-term and long-term plasticity. Sketch of the organic electrochemical transistor, formed by electropolymerization of **a** ETE-S and **b** ETE-PC in the transistor channel. **c** Paired-pulse facilitation (PPF) and paired-pulse depression (PPD) indices of the synapse operating in the short-term doping/de-doping mode. **d** Long-term potentiation and depression (LTP and LTD) achieved by stepwise electropolymerization and overoxidation of the channel, and long-term stability of a single state on electropolymerization showing retention times > 1000 s. **e** Spike-timing-dependent plasticity (STDP) versus time delay (Δ*t*) between presynaptic and postsynaptic terminal. **f** Representative voltage waveforms at Δ*t* = 0 and 90 ms, respectively. Images produced with permission from references: [124] Copyright 2019, Wiley–VCH; [125] Copyright 2022, Springer Nature

The combination of both short- and long-term plasticity in a single device allows for merged processing and memory functionalities resembling to non-von Neumann architectures. Fabiano et al. used dual-mode operations including electrochemical doping/de-doping and electropolymerization to modulate the conductance (Fig. 6) [124, 125]. The synaptic channel is formed by electropolymerizing self-doped conjugated monomer precursor (sodium 4-(2-(2,5-bis(2,3-dihydrothieno[3,4-b][1,4]dioxin-5-yl) thiophen-3-yl)ethoxy)butane-1-sulfonate (ETE-S) or (2-(2,5- bis(2,3-dihydrothieno[3,4-b][1,4]dioxin-5-yl)thiophen-3-yl) ethyl(2-(trimethylammonio)ethyl) phosphate) (Fig. 6a). The presynaptic input stimulates the accumulation of ions in the organic channel, resulting in a transient increase/decrease in channel conductance due to ionic doping/de-doping, which is analogous to the short-term synaptic function in biological synapses induced by the accumulation of Ca^2+^ and neurotransmitters in postsynaptic neurons stimulated by action potentials. This electrochemical redox is applicable to most electrolyte-gated organic synaptic transistors. The long-term increase in conductivity of the organic electrochemical synapse (OECS) was achieved by electropolymerizing additional ETE-PC in the channel by applying gate voltage pulses of − 0.6 V for a duration of 1 s. A total of 150 distinct states were demonstrated in the OECS, with state retention of more than 1000 s [125]. The changes in synaptic weight varied with the time delay between the presynaptic and postsynaptic spikes, reaching a maximum value when both spikes completely overlapped (Fig. 6b–f).

In biology, although the postsynaptic conductance disappears when stimuli are absent, the synaptic weight of the memory triggered by the stimuli cannot be quickly erased, which is an important merit of error tolerance in the brain [126]. The retention time of the synaptic weight is a reflection of the nonvolatile property of synaptic devices, which is analogous to the learning and memory functions of the brain. Of course, in biological nervous systems, the retention time are dynamic but varies depending on its roles and locations. For example, a long retention time is required for the memory and neuromorphic computing, which is inappropriate for sensory neurons. Therefore, the development of synaptic devices with different decay constants is of great importance to realize wide applications of neuromorphic devices. The decay time constant (*τ*) of neuromorphic devices can be calculated by employing the exponential decay function [123]. Taking the architecture and circuit of electrolyte-gated organic synaptic transistors as given, its synaptic functions are defined by the substrate, electrolyte composition, and the material of the organic channel, as summarized in Table 2.

**Table 2:** Summary of synaptic functions emulated by the state-of-the-art electrolyte-gated organic synaptic transistors

Electrolyte	Materials/ions in electrolyte	Energy consumption	Retention time	Conductance states	Synaptic functions	References
NaCl aqueous solution	PEDOT:PSS	–	< 1 s	> 2	STP, PPF	[32]
	PEDOT:PTHF	–	Few seconds	continuous	STP, LTP, PPF	[36]
	PEDOT:PSS at gate/PEDOT:PSS-PEI at channel	~ 10 pJ	100 s	512	STP, LTP, STDP	[33]
	P(ETE-PC)	–	> 1000 s	150	STP, LTP, STDP	[125]
	PBTTT:F4TCNQ at gate, and PBTTT at channel	–	–	> 250	STP, LTP	[105]
Ionic liquid	PDPP/EMIM:TFSI	sub-100 fJ	–	> 2	STP, LTP	[34]
	P3HT/EMIM:TFSI	0.06 fJ	Few seconds	> 40	STP, LTP, PPF	[39]
Ion gel	gNR-Bu/Na_3_Cit	2.19 pJ	> 600 s	> 6	STP, LTP, STDP	[98]
	p(g2T-TT)/EMIM:TFSI/PVDF-HFP	~ 80 fJ	Order of minutes	continuous	LTP, STP	[82]
	PEDOT:PSS/EIM:TFSI/PVDF-HFP	~ 2.7 pJ				
	P3HT/LiTFSI	–	Few seconds	> 2	STP, LTP, PPF	[127]

#### Influence of Architecture of the Synaptic Devices on the Synaptic Functions

**Fig. 7:**
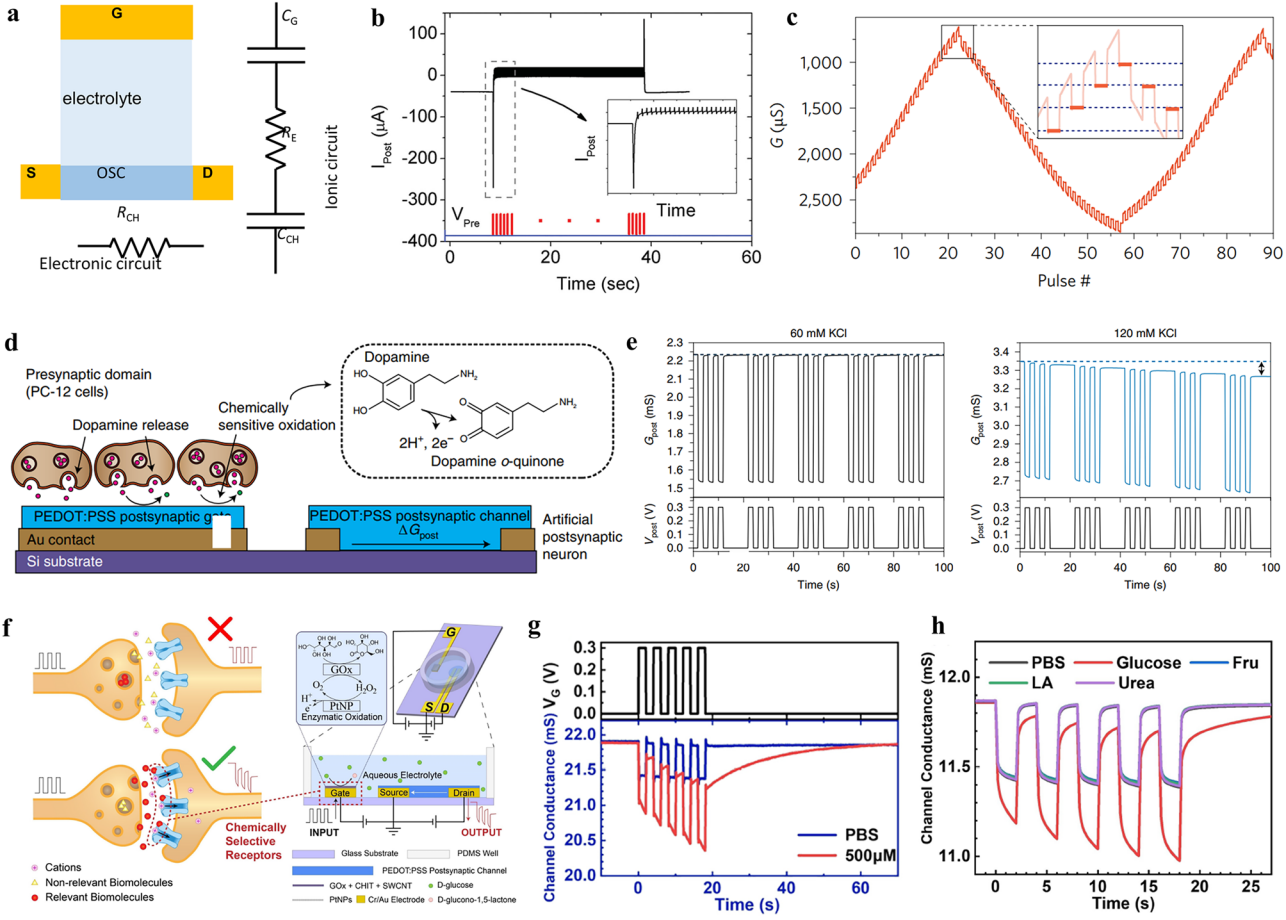
Influence factors on the status retention time. **a** Ionic and electronic circuit in electrolyte-gated organic transistors. *C*_G_, *C*_CH_, *R*_E_, and *R*_CH_ represent the capacitance of gate electrode, organic channel, resistance of the electrolyte and the organic semiconductors, respectively. **b** Adaption behavior of OECTs when a train of pulses is applied on the gate electrode. Reproduced with permission [32]. Copyright 2015, Wiley–VCH. **c** Long-term potentiation and depression of the electrochemical neuromorphic organic device controlled by the applied gate pulses. Reproduced with permission [33]. Copyright 2017, Springer Nature. Ions and biopolymer modulated neuromorphic devices. **d** Schematic of the neurotransmitter-mediated neuromorphic device. **e** Comparison of conductivity changes to postsynaptic voltage pulses coupled with PC-12 cells stimulated by a 60 mM KCl solution (left) and a 120 mM KCl solution (right), respectively. Reproduced with permission [4]. Copyright 2020, Springer Nature. **f** Schematic of a biological synapse containing several neurotransmitter receptors on the postsynaptic membrane and electrolyte-gated synaptic transistor device measured in aqueous solution. Channel conductance response to **g** different concentrations of phosphate buffered saline (PBS) solution and **h** different types of biomolecules: glucose, fructose, lactic acid, and urea. Reproduced with permission [128]. Copyright 2023, Wiley–VCH

For organic transistors operated in the electrochemical redox regime, such as organic electrochemical transistors, ions from electrolyte are injected or extracted from the organic semiconducting channel depending on the applied gate bias. The electrochemical doping/de-doping causes the modulation of the channel conductance. According to Bernard’s mode, OECTs consist of an ionic circuit and an electronic circuit (Fig. 7a) [118], where the ion circuit describes the flow of ions at the gate/electrolyte interface and electrolyte/organic channel interface and is treated as a resistor in series with a capacitor, while the electronic circuit describes the charge carrier drift in the organic channel driven by a local potential and is treated as a resistor. Unlike from the OFETs, where the channel thickness has no effect on the device performance, in OECTs, the volumetric gating indicates the critical role of the channel thickness on the device performance, such as the transconductance and response time. Accordingly, OECTs typically have a better amplification performance compared to other FET devices [25].

The response time of the device to the applied presynaptic pulse is related to the thickness of the channel or the distance between the presynaptic terminal and the postsynaptic terminals. Reducing the thickness of the channel could reduce the diffusion distance and thus improve the time response. As shown by Gkoupidenis et al., when a pulse train is applied, the OECT-based synaptic devices respond to the pulse by exhibiting depression and adapting to it, while the subsequent removal of the gate pulse resulted in a rapid recovery of the channel conductance to its original state, demonstrating its response in short-term nature (Fig. 7b). A battery-like electrochemical neuromorphic organic device was then developed by Burgt et al. consisting of a PEDOT:PSS-modified gate electrode and PEDOT:PSS partially reduced by PEI as the channel materials, as shown in Fig. 5a. The proton-doped PEI compensates the PSS via the application of a positive gate pulse, resulting in the de-doping of PEDOT and a consequent decrease in the channel conductance (Fig. 7c). As PEI effectively stabilized the neutral state of the PEDOT in the PEDOT:PSS/PEI channel and the electrolyte acted as a barrier for the electronic charge transport, the conductance states could be maintained for a while and the device was considered to perform in a nonvolatile operation [33]. The detailed description of electrochemical resistive memories can be found in the literature [24].

#### Influence of the Channel Materials on the Synaptic Functions

As mentioned above, the key factor in determining the retention time is the ion migration in the circuit under biasing the gate. For the EDL-controlled EGTs, ions from the interface could rapidly diffuse back to the bulk solution and reach the original distribution when the gate bias is removed, since the EDL is a few nanometers thin. Consequently, conductance switching processes require less than 1 ms [129]. In contrast, in the case of ion-permeable/thick organic transistors, the ions penetrating through the organic film induce electrochemical doping/de-doping of channel materials, so that the retention time can vary from a few tens of milliseconds to minutes, depending on the permeation depth [130]. The permeability of the organic semiconducting polymers utilized in the EGT is summarized in Table 1.

Reducing the channel thickness could decline the diffusion distance, resulting in a decrement of the retention time. In addition, the wettability of the semiconducting channel materials could tune the response behavior of the electrical devices. Side-chain engineering is a versatile method for modifying the processability of polymers. Giovannitti et al*.* utilized triethylene glycol side chains to replace the alkylated analogous with the same backbone, thus tailoring the operation of the electrolyte-gated transistors from the interfacial-doping OFET to the bulking-doping OECT [62]. In our recent work, a novel *n*-type conducting polymer PBFDO was developed, which exhibited high conductivity comparable to the typical *p*-type polymer PEDOT:PSS. However, due to the inferior wettability, the resulting OECT device suffered from a slow transient response, that is incapable of matching that of its p-type device counterparts, limiting its further application in the complementary circuit [65]. Recently, we incorporated hydrophilic PEG side chains into PBFDO to accelerate the ions penetration into the ionic circuit, and tuned its wettability by altering PEG contents, which significantly reduced the response time and yielded an inverter with high gains [131]. The microstructure of channel materials also exerts crucial influence on the plastic behavior [132]. Go and the colleagues demonstrated that the long-term retention of ion gel-gated P3HT transistors could be efficiently prolonged by controlling the microstructure of P3HT semiconductor. Thereinto, the highly crystalline P3HT could be achived by spincoating the polymer solution on the substrate precoated by a self-assemble monolayer. The increased crystallinity impedes ion migration and thus extend the retention time of a certain state [133]. In addition, an organic synaptic transistor operated in enhancement mode has a higher dynamic range compared to one operated in depletion mode, as the intrinsically high resistance enables efficient electrochemical gating [82].

#### Influence of Electrolyte on the Synaptic Functions

As a key element of the ionic circuit, the electrolyte used in the EGST determines the amount and rate of ion injection into the semiconductor channel, thus affecting the synaptic functions of the devices. The commonly utilized electrolytes for electrolyte-gated synaptic transistors are aqueous solutions, ionic gels, and ionic liquids. The sole requirement is that the ions in these media can rearrange in response to the external stimulation [62]. Aqueous electrolytes with a similar composition as physiological fluids are the ideal candidates for bioelectronics, facilitating the accurate mimicking of biological synapse functions. The organic channel materials in synaptic OECT devices can communicate with the external aqueous environment directly and the ionic and electronic coupling between the ions from electrolyte and the semiconducting polymer effectively tune the channel conductance within low gate bias ranges, favoring the construction of artificial synapses with low operation consumption [118].

The incorporation of Nafion membranes could extend the retention time mainly by slowing down the ion diffusion rate after the removal of the gate pulse [134]. Additionally, the ion concentrations and the type of ions affect the relaxation behavior of devices. In general, higher ion concentrations result in larger synaptic weight and faster responses due to stronger ion–semiconductor coupling resulting in faster doping/de-doping processes. Similar phenomena have been observed for low atomic mass ions (e.g., Na^+^) compared to high atomic mass ions (e.g., Mg^2+^, Ca^2+^) due to the faster ion migration [134, 135]. This process is analogous to the biological synapse, where higher synaptic strength could be stimulated when large amounts of Ca^2+^ and neurotransmitters are released to the postsynaptic neurons. Given the different nature of biological synapses, the chemical synapse should be taken into consideration in addition to the discussion on electrical synapses above.

In biological chemical synapses, the long-term connection between neurons is dynamically regulated by the local neurotransmitter activity [91]. Keene et al. developed a functional biohybrid synapse with a dopaminergic presynaptic domain generated by PC-12 cells and an organic synaptic transistor as the postsynaptic domain (Fig. 7d) [4]. Dopamine release via exocytosis by PC-12 cell is locally oxidized at the postsynaptic gate electrode, changing the charge states of gate electrode and induces the migration of ions between electrolyte and organic channel, thereby altering the channel conductance. Receptor-mediated phagocytosis and exocytosis of dopamine were also emulated by applying a postsynaptic voltage pulse while measuring in different concentration of aqueous KCl solution. It was found that only a high concentration of KCl could effectively stimulate conductance changes in the postsynaptic channel due to the high release rate of dopamine (Fig. 7e). Additionally, the specific combination between receptors and neurotransmitters is of fundamental importance in the modulation of synaptic functions, since the opening of ion channels and the reflux of ions across the postsynaptic membrane occur and trigger the long-term modulation memory effect only when the specific combination is present, and vice versa. Xu et al*.* mimicked the selective modulation of the synaptic plasticity by specific enzymatic reactions between glucose and its corresponding enzymes and revealed that only the introduction of glucose could stimulate long-term conditioning of the channel conductance (Fig. 7f–h) [128]. Therefore, it is fundamental to considered the species of the aqueous electrolytes for the development and operation of electrolyte-gated synaptic transistors.

**Fig. 8:**
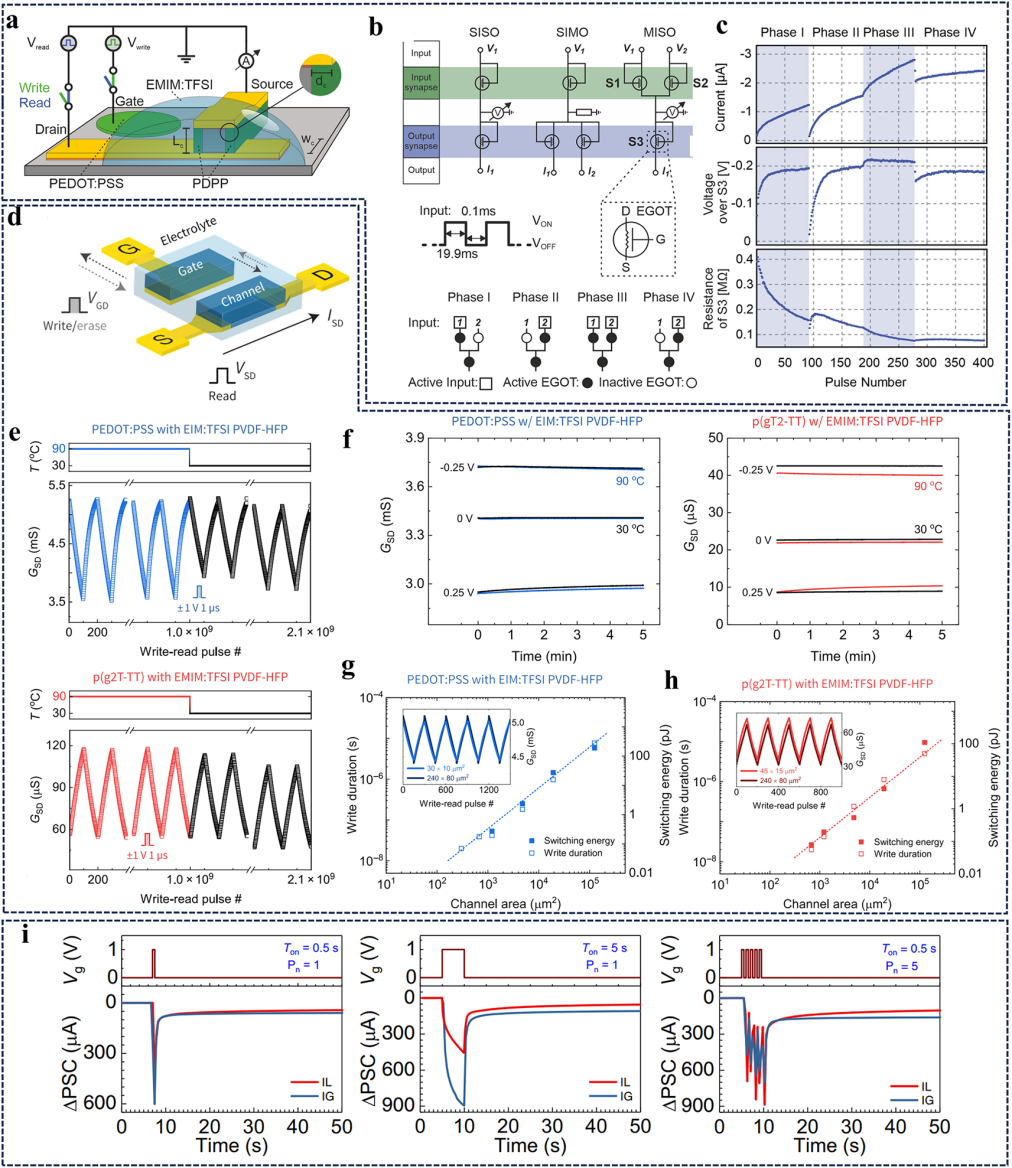
Influence of electrolyte on the synaptic functions of EGOST. **a** Structure of ionic liquid-gated vertical organic transistors. **b** Tested circuit configurations with the three marked synapses: single-input single-output (SISO), single-input multiple-output (SIMO), and multiple-input single-output (MISO). **c** Current output, voltage drop, and resistance across the output organic transistors at different phases displayed in **b**. Reproduced with permission [104]. Copyright 2022, American Chemical Society. **d** Schematic structure of ion gel-gated electrochemical memristors. **e** Channel conductance response to the write–read events occurs at 90 (blue/red) and 30 °C (black) together with the application of ± 1 V/1 µs pulses. **f** State retention of device measured at 30 and 90 °C, respectively. Dependence of switching speed and energy consumption of **g** PEDOT:PSS EIM:TFSI/PVDF-HFP-based device and **h** p(g2T-TT) EMIM:TFSI/PVDF-HFP-based device on the channel area under < 1 μs write–read cycles. Reproduced with permission [82]. Copyright 2020, AAAS. **i** Comparison in changes of synaptic weight for devices separately gated by ion gel and ionic liquid by varying the amplitude, duration, and numbers of gate pulses. Reproduced with permission [37]. Copyright 2023, Springer Nature

Noteworthy, ionic liquids considered as room-temperature molten salts are excellent substitutes for aqueous solution in EGOT. Their superior electrochemical stability, wide electrochemical window, optical transparency, low volatility, high ionic conductivity, as well as high thermal stability (up to 350 °C) make them an obvious choice [136–139]. The formation of an EDL at the ionic liquid/semiconductor interface under a voltage bias results in the generation of a high electric field, which in turn induces a high carrier density/mobility. This enables the device to operate at a significantly low voltage [140]. Weitz’s group have developed vertical organic transistors with 1-ethyl-3-methylimidazolium bis- (trifluoromethylsulfonyl)imide ([EMIM][TFSI]) as the electrolyte and P3HT as the semiconductor. These exhibited an on/off current modulation ratio of up to 10^8^ and sub-100 fJ energy usage per synaptic event when used as a memristive device (Fig. 8a) [34]. In their further work, they fabricated nanoscopic vertical transistors with diketopyrrolopyrrole-terthiophene donor–acceptor polymer (PDPP) as the channel material, achieving a switchable voltage down to − 1 mV. This is a promising development for low-energy memory devices and demonstrates a history-dependent information transfer of interconnected systems with multiple inputs and outputs (Fig. 8b, c) [104]. Furthermore, ionic liquid is an excellent dielectric medium for OECTs, significantly improving the devices response rates and reaching the maximum transistor gain at lower gate voltages, which is ideal for low energy consumption [141]. The most commonly used ionic liquid in the synaptic transistors is 1-ethyl-3-methylimidazolium bis-(trifluoromethylsulfonyl)imide ([EMIM][TFSI]). The charge carrier mobility of organic semiconductors is dependent on the ionic liquid’s components [140]. It is therefore important to optimize the ionic liquid in order to achieve the desired device performance and the synaptic functions.

The versatile merits of liquid electrolytes are undeniable. However, its liquid nature makes it unsuitable for use in flexible devices or circuits. To this end, ion gels have been developed. They provide mechanical compatibility with polymers and swift switching responses due to the ionic conductivity similar to ionic liquids, mitigating liquid leakage and forming an EDL for an operation of the devices at low voltages. Solid electrolyte are also a promising alternative to liquid electrolytes especially for the flexible devices. The first ionic liquid-based gel for EGOTs was developed by Lee et al. [142] by blending 1-butyl-3-methylimidazolium hexafluorophosphate ([BMIM][PF6]) with a tri-block copolymer. A summary of polymers and ionic liquids used for ion gel transistors can be found in the literature [143]. Currently, the P(VDF-HFP):[EMIM][TFSI]-based ion gel is the most widely utilized gel in neuromorphic devices due to its easy transfer, high tension, structural stability, and ease of operation through a “cut and paste” method. Melianas et al. investigated the influence of temperatures on the synaptic functions for P(VDF-HFP):[EIM][TFSI] and P(VDF-HFP):[EMIM][TFSI] ion gel-gated organic transistors with the corresponding utilization of PEDOT:PSS and P(g2T-TT), respectively (Fig. 8d) [82]. The all-solid synaptic transistors were stable under > 10^9^ read/write events and nearly temperature-independent operation even at the high temperature of 90 °C and exhibited tunable conductance states as well as high signal-to-noise ratio above 100 (Fig. 8e). The PEDOT:PSS/[EIM][TFSI] PVDF-HFP-based device exhibited an estimated state retention of around 1 min at both 30 and 90 °C, while P(g2T-TT)/[EMIM][TFSI] PVDF-HFP exhibited the value of > 5 min at 30 °C and around 1 min at 90 °C (Fig. 8f). The high switching speed (~ 20 ns) and low energy (~ 80 fJ) per write process make ion gel-based synaptic transistors promising devices for the implementation in artificial neural networks (Fig. 8g, h). A comparison of ionic liquid and ion gel in tuning the synaptic functions of synaptic transistors showed that ion gel-gated devices achieved a higher synaptic weight and retention time compared to ionic liquid-gated devices, mainly due to the slow redistribution of ions within the networked polymer matrix after the removal of electrical stimuli (Fig. 8i) [37]. Some other electrolytes, such as polymer electrolytes and polyelectrolytes have shown an excellent capacitance performance and exhibit slow ion migration [129, 144], enabling their applications in emulating long-term plasticity.

#### Influence of Mechanical Deformation on the Synaptic Functions

In biology, synapses are soft and stretchable, allowing them to easily adapt to various forms of mechanical deformation [145]. The development of flexible and stretchable synaptic devices is of paramount importance for the conformal integration with biological tissues and the realization of neurological functions in soft machines or other applications, such as artificial cognitive skins, artificial organs, and neuroprosthetics [41, 146–149]. Stretchable organic synaptic transistors generally require a gate/source/drain electrode, electrolyte, organic semiconductors, and substrates, and all of them need to exhibit excellent mechanical conformability but also to withstand large deformations and multiple stretching cycles. The commonly utilized substrates for stretchable transistors are elastomers such as poly(dimethylsiloxane) (PDMS) [150], polystyreneblock-poly(ethylene-ran-butylene)-block-polystyrene (SEBS) [151], or thermoplastic polyurethane (TPU) [152]. The electrode materials need to satisfy high mechanical strength, high conductivity similar to the commonly used metal electrodes or indium tin oxide (ITO), as well as low contact resistance to facilitate the injection and extraction of charge carriers into the channel materials and from the electrodes, respectively. To date, several highly conductive materials including carbon nanotubes [127, 150], Ag nanowires [35, 146], and conductive polymers (e.g., PEDOT:PSS) [153] have been successfully employed as compliant electrode materials to fabricate stretchable synaptic transistors. Additionally, the flexibility and stretchability of ion gels, mentioned as highlight features in the last section, make them promising candidates as electrolytes for stretchable synaptic transistors. The key component of stretchable synaptic transistors are flexible (semi)-conducting polymers which have been prepared based on the following strategies: deposition of semiconducting polymer films on the prestretched substrates, blending conducing polymers with the aforementioned elastomers, or synthesizing intrinsically stretchable semiconductors. The progress on the development of functional layers for stretchable synaptic transistors is detailed in the literature [154].

**Fig. 9:**
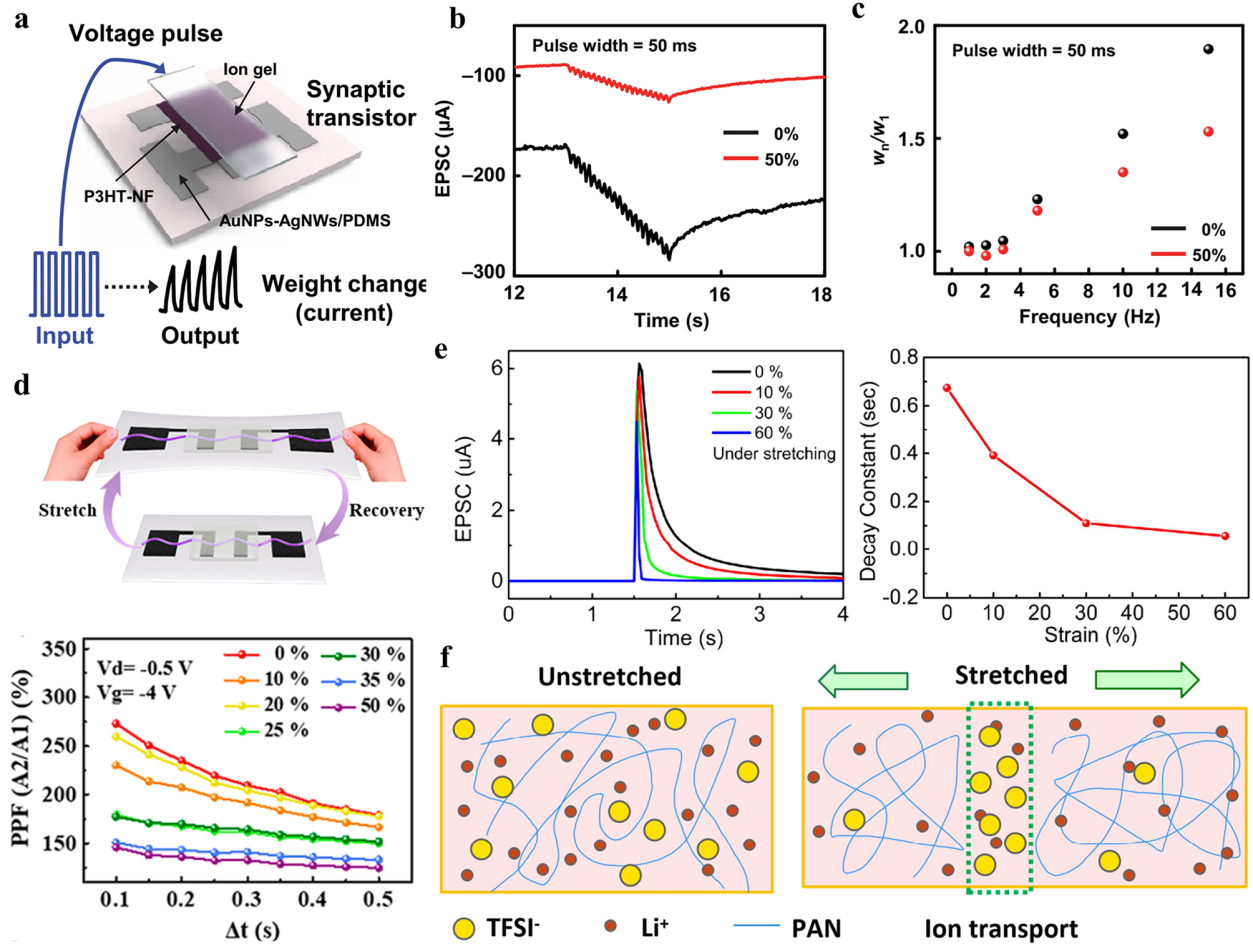
Synaptic functions emulated by stretchable electrolyte-gated organic transistors. **a** Schematic structure of stretchable synaptic device stimulated by multiple presynaptic inputs. **b** Changes in excitatory postsynaptic current (EPSC) under different mechanical strains of 0% and 50% stimulated by 20 successive presynaptic pulses. **c** Evolution of *w*_n_/*w*_1_ of the stretchable synaptic transistors with respect to the applied pulse frequency under mechanical strains of 0% and 50%. Reproduced with permission [35]. Copyright 2019, AAAS. **d** Schematic illustration of the P3HT/PEO NW-based device experienced repeated stretching and recovery (top). PPF index changes versus the time interval between two successive spikes at different tensile strains (bottom). Reproduced with permission [155]. Copyright 2022, American Chemical Society. **e** EPSC and synaptic decay constants as a function of strains. **f** Schematic of ion transport in ion gel of stretchable synaptic transistors. Reproduced with permission [127]. Copyright 2020, Elsevier

The effect of mechanical deformation on the basic synaptic functions including the PPF and EPSC is shown in Fig. 9. Under a train of presynaptic pulse, the changes in synaptic weight and PPF are much smaller for the stretched devices than for the unstrained ones, and the removal of spikes causes significant changes in the long-term behavior such as memory, especially in the case of large mechanical deformations (Fig. 9a–d) [35, 155]. Additionally, the decay constants decreased significantly with strain, demonstrating a rapid response to stimulations (Fig. 9e) [127]. Noteworthy, ion gels are used as the electrolyte for most stretchable transistors, although ions are normally confined to the polymer matrix. However, the application of mechanical deformation, especially in the case of large deformation, results in the disentanglement of polymer chains and the reduction of the interrelation of the polymer networks, thus enhancing the ion transport in the gel and accelerating the resulting rearrangement of ions under or after the presynaptic spikes (Fig. 9f) [127].

#### Influence of Stability on the Synaptic Functions

Although EGOTs hold great promise as artificial synapse to emulate versatile plastic functions, the electrochemical mechanism and organic materials of EGOTs makes the devices susceptible to parasitic chemical reaction in ambient conditions [156], which may cause adverse effect on EGOTs and thus reduce the device lifetime. Addressing stability challenges is critical for their successful implementation in practical scenarios, where precise modulation of the plastic behavior is required for neuromorphic computing or biointerfacing [157]. The typical manifestation in the instability of EGOTs is the current drift, either increase or decrease, over time.

Regardless of the biocompatibility and easy of fabrication, organic semiconductors are susceptible to aqueous environment, which can degrade the charge transport properties of the organic materials. Kenne and the colleagues demonstrated that the electrochemical neuromorphic organic devices programmed in air exhibited a limited conductance range and a significant decay of the state under reduced potentials due to the chemical reaction of PEDOT:PSS with oxygen gas, which could be avoided when operated in N_2_ atmosphere. By replacing the aqueous KCl electrolyte by a Nafion thin-film solid-state electrolyte, there was a prominent increase in the absolute conductance of the device and the state retention improved by an order of magnitude by operating in an inert environment [158]. Apart from the chemical reaction, water molecules diffused into the polymer bulk cause torsional and other defects in the polymer backbone [159], as well as irreversible microstructural changes, thus affecting the long-term stability of devices. Our previous work found that the significant swelling behavior of n-type conducting polymer PBFDO occurred when electrochemically doped in diluted NaCl aqueous solution due to the penetration of Na^+^ accompanied by plenty of water molecules into the channel material, which potentially hampered the transport of charge carriers, consequently diminishing the OECT performance [64]. Moreover, high ionic strength of electrolyte, such as buffers or saline solutions utilized in biosensing, introduce even more pronounced instabilities [160].

In addition, Simatos and the colleagues demonstrated contaminations introduced during the fabrication process of neuromorphic devices, such as plastic laboratory consumables and un-cross-linked PDMS, can affect the baseline of transistors and thus yield artifacts for a biosensing event. Meanwhile, galvanic corrosion of the metal contacts is another source of device degradation. The adhesion layers (Ti or Cr) and the active metal layer (Au) may form an electrochemical cell when exposed to an aqueous solution, resulting in a rapidly decreasing ON channel current [157]. Additionally, by-products generated during potential electrochemical reactions conducted in “static mode” and air bubbles formed when integrated the neuromorphic devices with microfluidic channels may disrupt the operation stability and the resultant plastic behaviors [157, 161].

Research efforts focusing on enhancing material durability, optimizing electrolyte composition, controlling the environments (operating in inert environment, e.g., a nitrogen gas purged glove box) as mentioned above, encapsulation [162], and exploring advanced device architectures will be vital in overcoming these issues. Diketopyrrolopyrrole (DPP)-based polymers have been widely utilized to realize enhanced EGOT performance by side-chain engineering and conjugated backbone chemistry. Zheng and the colleagues modified the donor–acceptor semiconductor DPPTT through covalent functionalization of fluoroalkyl chains onto the film surface to form densely packed nanostructures, significantly improving the stability of the polymer against moisture, oxygen, and light [163]. Sung and the colleagues controlled the synaptic functions of DPP-based EGOTs through altering the copolymerization ratio of side alkyl chains of 2-octyldodecyl (-OD) and 2-decyltetradecyl (-DT). They found that device based on high content of DT exhibited rapid decrease in current even the application of high voltage and multiple stimulation, with the relaxation time to be 0.011 s, as well as obvious linear property. In contrast, the EPSC exhibited a gradual decrease to its initial value with the relaxation time of 1.58 s and a more idealized long-term linear potentiation/depression behavior with increasing the OD content [164].

## Biophysical Realizations of Electrolyte-Gated Organic Transistors

In humans, sensory systems such as olfaction, vision, hearing, taste, and touch are made up of sensory neurons. Signals generated by the specific response of receptors in these sensory neurons to stimuli are transmitted to other neurons in the vicinity of the brain through the diffusion of neurotransmitters across synapses to realize the signal perception [165, 166]. The recent advancement of versatile electrolyte-gated organic synaptic transistors (EGOSTs) and digitalization process of sensory devices, which enable the emulation of biological perceptional functions and seamlessly integrate digital technology with the biological counterparts and environments [76], caters to the growing interest in constructing biomimetic perceptional systems and neurons.

### Artificial Olfactory System

**Fig. 10:**
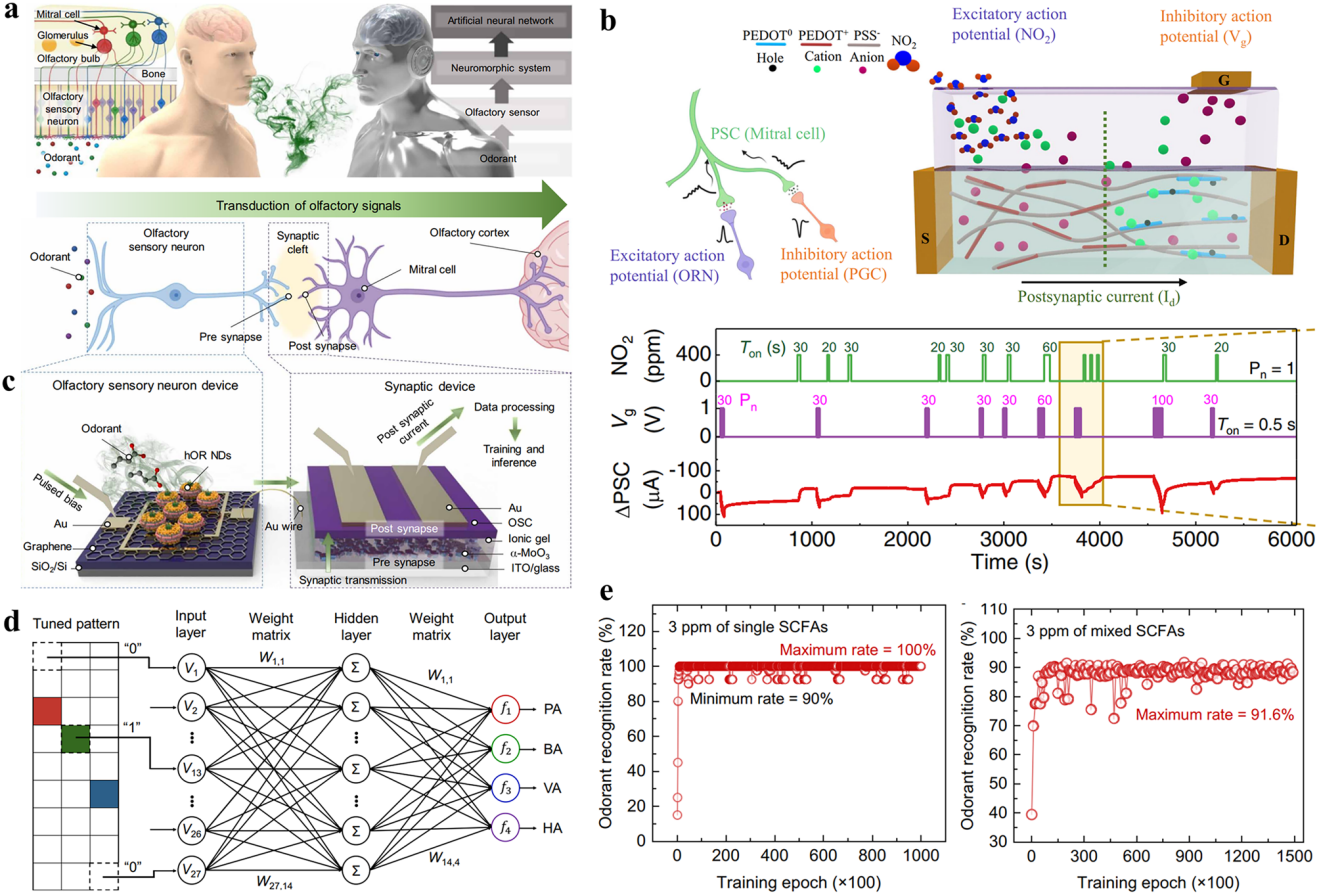
EGOSTs as artificial olfactory system. **a** Schematic illustration of the biological olfactory system (left) and the artificial olfactory system (right). Reproduced with permission [168]. Copyright 2024, AAAS. **b** Chemosensory neuronal synapse for recording excitatory and inhibitory functions triggered by the electrical and chemical (gas molecules) stimulation. Reproduced with permission [37]. Copyright 2023, Springer Nature. **c** Structural overview of the olfactory sensory neuron device and organic synaptic device. **d** A custom-designed artificial neural network (ANN) including 27 input neurons, 14 hidden neurons, 4 output neurons, and 27 × 14 × 4 artificial synapses connecting the neurons. **e** Single-odorant recognition accuracy during 100,000 training and inference cycles and mixed-odorant recognition accuracy during 150,000 training and inference cycles

As one of the oldest functions we possess as animals, olfaction plays a vital role in our daily lives, as the odors generated by volatile chemicals could be detected by the sense of smell long before the sense of sight, and served as a survival cue. Typically, humans can distinguish up to 10,000 odors, ranging from the sweet aroma of fruit to the foul smells of sulfurous compounds. Olfactory receptor neurons, which are sensory neurons with a single dendrite and many cilia exposed on the surface of the epithelium, are vital in discriminating odor targets (Fig. 10a). When chemical molecules dissolve in the mucus of the olfactory epithelium, they could bind specifically to the olfactory receptor protein on the cilia, leading to the depolarization of the membrane within the dendrite, subsequent firing an action potential, and release of neurotransmitters from sensory neurons to afferent neurons once the depolarization exceeds the threshold potential [167].

Given the specific binding of molecules to the receptors on the sensory neurons, the implementation of artificial receptor sites is of great importance in the development of highly sensitive artificial olfactory devices. Chouhdry et al. fabricated an artificial chemosensory neuronal synapse based on a chemoreceptive ion gel-gated electrochemical transistor. N_2_O_4_ molecules, formed by the dimerization of the target molecule NO_2_ formed π–π interactions with the [EMIM]^+^ cations, which presumably caused the injection of [TFSI]^–^ anions into the channel to interact with PEDOT^+^ and the resulting increase in the postsynaptic conductance (PSC) due to the electrochemical doping (Fig. 10b) [37]. In addition, the PSC is easily modulated by the gas concentrations, and unlike the rapid recovery of the PSC triggered by electrical pulses, the increase in channel conductance is maintained for hundreds of seconds after the termination of the gas exposure. This allows for long-term memory, probably due to the strong interaction between the solvated complex of N_2_O_4_ and [EMIM]^+^ cations that prevents the diffusion of anions back into the gel.

Considering the ability of the mammalian olfactory systems to discriminate between a wide range of odorants and their mixtures, it is not sufficient for neuromorphic olfactory sensors to detect only a single component, since the gas composition in the atmospheric environment is extremely complicated. Therefore, the development of an artificial olfactory system with high specificity and accuracy is fundamental to emulate natural sensory systems. Song et al. recently developed an artificial olfactory system by synergistically integrating human olfactory receptors with artificial synaptic devices. In addition to an ion gel-gated organic transistor, a graphene extended-gate device is used as the sensory neuron device, on which the hOR NDs could be incubated (Fig. 10c). The exposed odorant interacted with the receptors, resulting in a detectable change in the device resistance, which was then transmitted to the presynaptic electrode of the synaptic transistors, driving the migration of ions in the ion gel and the resulting modulation of the channel conductance in the postsynaptic terminal [168]. Due to the specific binding interactions between the receptor and the odorants, the artificial olfactory system could identify odorants precisely at the chain length level. In addition, an artificial neural network (ANN) was established, which is of great importance for converting sensory inputs into digital data that can be processed and analyzed by the digital systems [76]. The training and inference simulation using an ANN demonstrated high detection accuracy for both single- and mixed-odorant recognition (Fig. 10d, e) [168].

### Artificial System for Visual Perception

**Fig. 11:**
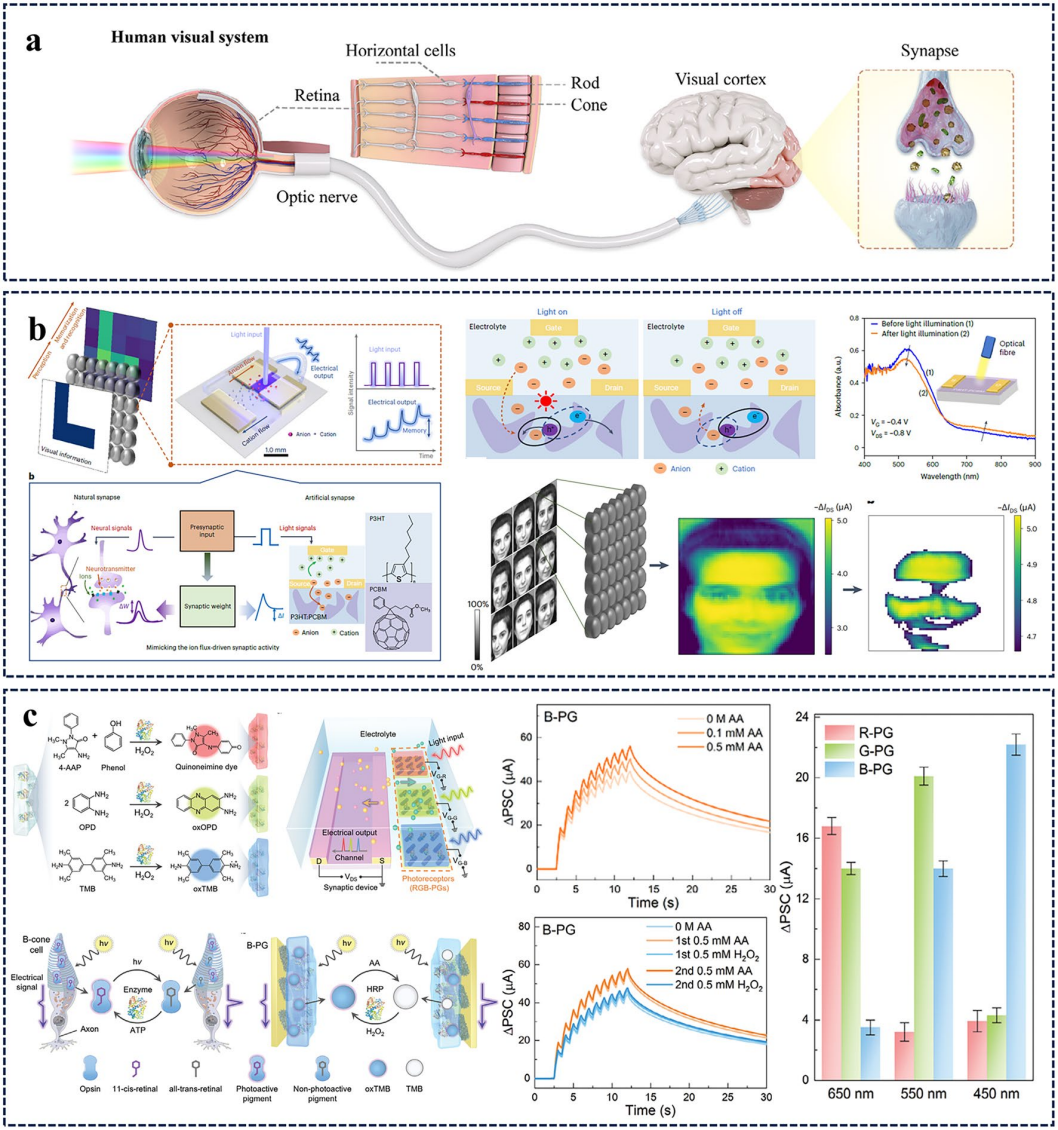
Artificial visual system. **a** Schematic illustration of the human visual system composed of the retina, optic nerve, and visual cortex. Reproduced with permission [169]. Copyright 2024, Wiley–VCH. **b** Schematic illustration and the working mechanism of photon-modulated electrochemical doping for artificial retina that is capable of image perception, memorization and recognition. Reproduced with permission [170]. Copyright 2023, Springer Nature. **c** Structural illustration of “multi-color” hydrogel-based photoelectrochemical retinomorphic synapse and the application for specific color perception and biomolecule-mediated synaptic plasticity. Reproduced with permission [166]. Copyright 2024, Wiley–VCH

In biology, more than 80% of the external information perceived by the human brain is obtained through the visual systems. Visual sensation is realized through the capture of photons by the sensory organ eyes, and the perceptible wavelength of the human eye ranges from 400 to 700 nm. The key element for visual perception in the sensory system is the retina, which is the light-sensitive region at the back of the eye where the photoreceptors are located. Before converging on the retina, light rays pass through the cornea, pupil, and lens (Fig. 11a). The photoreceptors are responsible for detecting the photons and converting them into electrical signals by triggering the release of neurotransmitters. In addition to the photoreceptors, there are four other types of cells located in the retina: bipolar cells, ganglion cells, horizontal cells, and amacrine cells. The latter two cells are responsible for the lateral communication between the neuron layers and the axons of the ganglion cells bundle together to form the optic nerve, which projects to the visual cortex in the brain accompanying with transmitting the visual information.

The electrolyte-gated organic transistor, with its low operating voltage and chemical–ionic–electronic coupling, soft nature, and biocompatibility, is a promising candidate for emulating visual perception, since ions carry the electrophysiological signals in the retina, while the signal transmission is governed by biochemicals. A core element that enables the response to light is the photoelectric material. The last decade has witnessed the robust development of light-absorbing materials for optoelectronic synapses [171, 172], including organic semiconductors [173], perovskites [174], metal oxides [175], and low-dimensional materials [175]. The photoactive material could either be the active material in the postsynaptic channel to directly modulate the channel conductance, or located at the presynaptic terminal to induce the ion flow and further tune the synaptic weight under the irradiation of light. Chen et al. developed a light-gated OECT by incorporating the photoactive material [6, 6]-phenyl-C61-butyric acid methyl ester (PCBM) into the organic semiconductor P3HT as the channel material (Fig. 11b). The donor–acceptor heterojunction was introduced to generate charge carriers under light stimulation, which was accompanied by anion transport from the ionic liquid electrolyte for charge compensation, resulting in the increase in the drain current. The photonic response is strongly related to ion diffusion. Due to the presence of anions around the doped P3HT, the charge recombination processes decayed after the removal of light stimuli, contributing to the nonvolatile memory characteristics of the optoelectronic synaptic device [170]. Human memory can normally be formed by rehearsal events: learning, forgetting, and relearning. The nonvolatile characteristics of the device enable the long-term plasticity and learning experience behavior. To process and memorize the optical information simultaneously and realize artificial visual systems for image perception and memorization, the device array with high-density transistors has been successfully used for high-resolution face recognition when the transient photonic current was beyond the decision-making condition (Fig. 11b).

Another promising strategy, in addition to the incorporation of photoactive materials into the organic channel, is the engineering of the photoelectrochemical conversion at the presynaptic gate electrode. For example, Hu et al. used CdS quantum dots as photoactive materials, which were deposited on ITO electrodes coupling with a sandwich immunoassay against human IgG. The formation of the sandwich immunocomplex, the release of Ca^2+^, and the gelation of the hydrogel/graphene oxide simultaneously altered the photon absorption and interfacial mass transfer efficiency of the quantum dot/ITO presynaptic terminal. This led to a substantially inhibition of the photo- and current response in the PEDOT:PSS channel [69]. Color discrimination in the human visual system is realized by three types of cone cell photoreceptors in the retina, which are separately sensitive to the primary colors of R, G, and B [176]. In the work performed by Hu et al., a photoelectrochemical transistor was developed with primary color perception by incorporating a color-tunable hydrogel on the photoactive material Bi_2_S_3_-modified gate electrode (Fig. 11c). The synergistic effect between the color-tunable hydrogel and the Bi_2_S_3_ with broad-spectrum absorption endowed the device with color recognition in the electrolyte without the additional application of gate pulse. In addition, the biochemical-driven photoelectric conversion of the cone cell in the human visual system was emulated by modulating the hydrogel color with a reversible enzyme-catalyzed reaction between oxidized 3,3′,5,5′-tetramethylbenzidine (oxTMB, blue sphere in Fig. 11c) and TMB (white sphere). The chemical–ionic–electronic signaling of the EGOTs demonstrates their great potential as fundamental building blocks in artificial visual perception systems and in resembling biological visual systems.

### Artificial Auditory System

Sounds are generated by the vibration of an object with a certain frequency and amplitude, which periodically changes the air pressure. In the biological auditory system, the mechanical vibration is transmitted from the outer ear, via the middle ear, to the inner ear and converted into electrical signals to trigger auditory nerve impulses by the Corti in the cochlea. The key structure in the vertebrate hearing is the hair cell, which is embedded along the inner surface of the organ of Corti. Hair cells are not neurons and can be divided into inner hair cells and outer hair cells. The inner hair cells are responsible for sending the majority (> 95%) of auditory signals from the cochlea to the brain for processing by releasing neurotransmitters (glutamate) at the synapse with the spiral ganglion cell, while the outer hair cells act as vibration amplifiers to increase the mechanical deformation of the inner hair cells, thus ultimately modulating the synaptic strength.

In addition, accurate sound localization is essential in everyday life for information exchanges, risk avoidance, etc. Sound localization is achieved by detecting interaural time difference (ITD), which is virtually analogous to the spatiotemporal information processing of the human brain and involves neural networks with immense connectivity [54]. Traditional silicon-based neuromorphic circuits usually suffer from limited connections between different devices due to the predefined physical wiring network [177, 178]. Electrolyte gating provides advantages in establishing complex connections between individual devices. Taking advantage of these connectivity, Liu et al. constructed an artificial neural network consisting of two fully connected transistors established by the strong capacitive coupling effect of ionic liquid to simulate the function of sound azimuth detection [39]. The two transistors were considered as the left and right ear sensing neurons, respectively, and their difference in the postsynaptic current was utilized to associate with the sound azimuth.

**Fig. 12:**
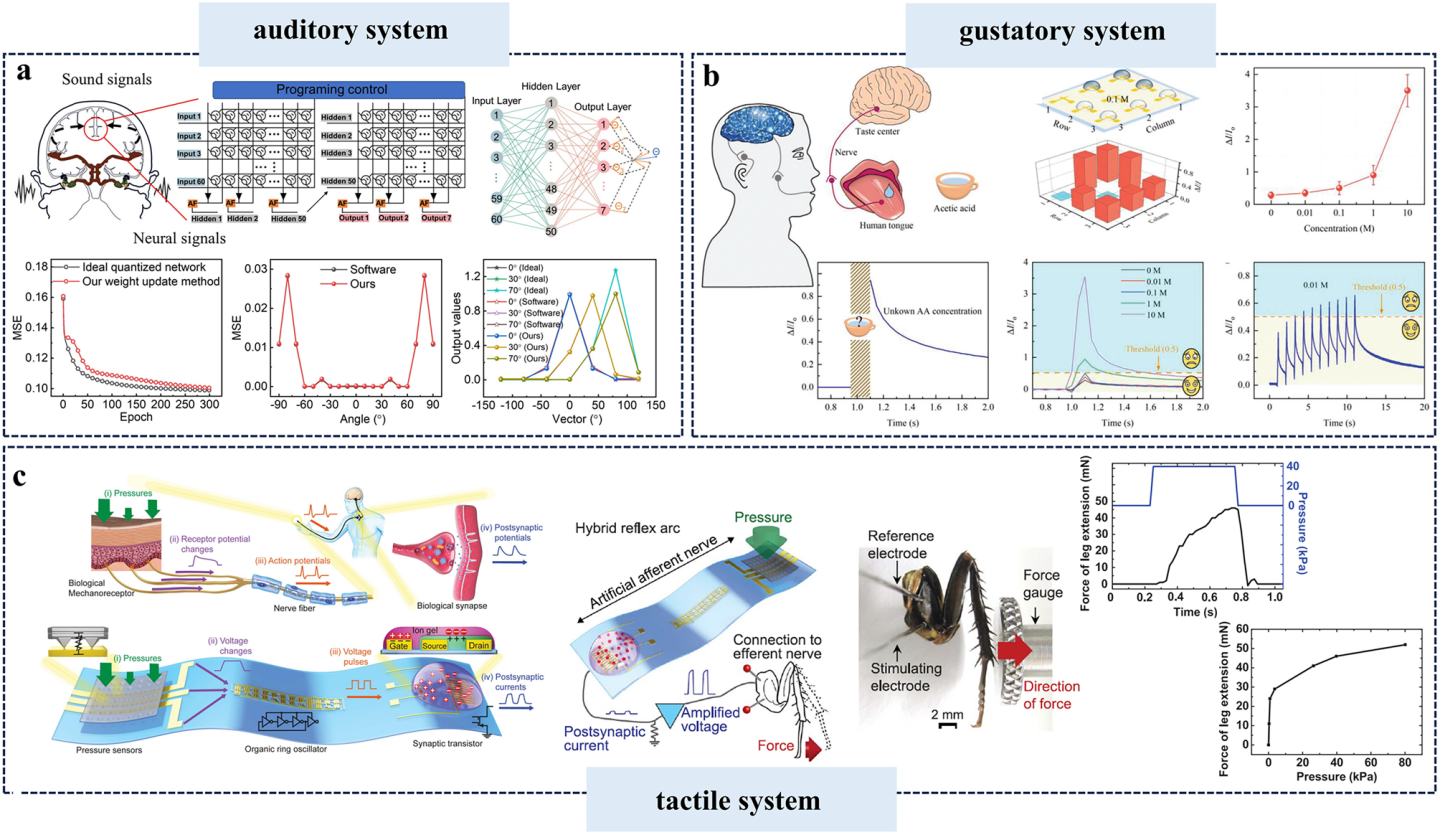
Applications of electrolyte-gated organic synaptic transistors (EGOSTs) as artificial perception systems. **a** Artificial auditory system. A three-layer artificial neural network structure for sound localization. Reproduced with permission [179]. Copyright 2023, AIP Publishing. **b** Artificial gustatory system. Artificial tongue for acetic acid detection. Reproduced with permission [39]. Copyright 2022, Wiley–VCH. **c** Artificial tactile system. Artificial afferent nerve formed by integration of pressure sensors, an organic ring oscillator, and a synaptic transistor and its application as reflex arc to stimulate biological efferent nerves and muscles to initial movement. Reproduced with permission [41]. Copyright 2018, AAAS

To further realize accurate sound localization, an artificial neural network was required that used the output neurons to predict the direction of the sound source, thereby significantly improving the detection accuracy. To this end, Xu et al. established a three-layer artificial neural network based on ion gel-gated organic synaptic transistors with P3HT as the channel active material. The detected sound signals were converted to the frequency domain, quantized to 16 levels, and then decomposed into values corresponding to angles ranging from − 120° to 120° (Fig. 12a) [179]. A conductance-controlled weight update method was used for the sound localization simulation calculations. The experimental results were in good agreement with the ideal quantized network, indicating the potential of electrolyte-gated organic synaptic transistors for neuromorphic simulation computation. However, the current artificial auditory systems based on synaptic transistors are only able to detect the location of a single sound source and lack noise robustness acoustic signals from noisy environments, which requires massive integration and computation. Therefore, miniaturization of synaptic transistors is essential to achieve high transistor density.

### Artificial Gustatory System

The human gustatory system detects the foods put in our mouth and provides us with information we need to make the right choices on our food to stay healthy. As the second chemosensory system beside the olfactory system, the human gustatory system can distinguish five primary tastes including salty, sweet, sour, bitter, and umami. This distinction is based on specific interactions between taste receptors expressed by taste cells in the taste buds and the chemicals in food [180]. When the receptor cells are stimulated by taste creating chemicals, only two neurotransmitters (ATP and serotonin) are released to transmit information to the central nervous system. Serotonin is released by the salt and acid taste cells, which are mediated by ionotropic taste receptors and depend on the ion channel to depolarize the cell. ATP is released in sweet, bitter, and umami taste cells in virtue of G-protein coupled receptors (metabotropic taste receptors) and second messengers that open ATP channels. Furthermore, each taste cell and the related afferent taste axon is specific to the type of taste, so a correct connection between the cells in the buds and taste neurons is fundamental.

Electrolyte-gated transistors hold great promise in mimicking the chemosensory gustatory system. The channel materials can interact with the ions in the electrolyte either by the formation of an EDL or by electrochemical redox processes. The human tongue directly contacts with the environment, so the targeted chemical substance can be absorbed by the electrolyte or interact with the presynaptic electrode. Liu et al. developed an artificial tongue based on vertical organic transistors for acetic acid (AA) discrimination with an ionic liquid encompassing different concentrations of AA as a thin salivary layer on the tongue [39]. The changes in channel current were utilized to discriminate the acidity of the target samples, which increased with the AA concentration and reached to a threshold at 0.1 M acid content (Fig. 12b). An electric pulse was applied to emulate the memory function in the artificial tongue. The continuous stimulation of electric pulses at a constant AA concentration lower than the saturation caused the expression of a “pain feeling”, which is analogous to the behavior of a human tongue repeatedly licking acetic acid.

The receptor cells in the human tongue are able to convert chemical stimuli into ionic signals after specific recognition by ion channels. Inspired by the selectivity of ion channels, Zhang et al. constructed a nanofluidic membrane to implement the selective cation transport in OECT devices [181]. The selective membrane was prepared with 2D metal–organic frameworks through the coordination reaction between tripodal bridging 2, 3, 6, 7, 10, 11-hexahydroxytriphenylene (HHTP) ligands and Cu^2+^ cations. The negatively charged nanofluidic membrane electrostatically attracts cations and repels anions in the analyte solution. This provides sufficient transport pathways for cations, facilitating their migration into the organic channel, de-doping of PEDOT, and reducing the channel current. The nanofluidic OECTs exhibit a distinguishable response to cations of different sizes due to the steric hindrance effect of the nanofluidic membranes [181].

The current study concerning artificial gustatory systems based on electrolyte-gated transistors is still in its infancy. Related research mainly focuses on the detection of acid or salts, which are rich in ions and have no specificity. In contrast, the human tongue is a smart system. It can not only perceive and distinguish specifically different tastes, but also recognize temperature variations from food or pressure from teeth. To achieve the same level of functionality as a biological gustatory system, the integration of neuromorphic transistors with other functional sensory elements is essential.

### Artificial System for Tactile Sensing

The human tactile system comprises a series of mechanoreceptors that enable us to perceive touches (e.g., pressure, vibration, stretch, and motion) on the skin. These mechanoreceptors are primary neurons and branch into two directions. One branch extends toward the skin surface and the other one travels toward the spinal cord. Unlike the voltage- or neurotransmitter-mediated mechanism in other perception systems, the ion channels in the somatosensory system in the tactile system are mainly gated by mechanical distortion or stretch of the cell membrane. The mechanical forces induce the opening of ion channel, enabling the influx of cations into the neuron and firing action potentials, which is finally transmitted to central nervous system through the spinal cord to form tactile sensation.

Tactile reception is a complex process. It involves collecting information from multiple tactile mechanoreceptors, transducing the mechanical into electrical signals, and conveying this information to biological efferent nerves. Current tactile sensors can be divided into four main categories based on their conduction mechanism: resistive, capacitive, piezoelectric, and triboelectric [182–185]. Electrolyte-gated organic transistors, which act as artificial synapses, can execute the tasks of signal conversion and neuromorphic operations to emulate biological functions such as learning and memory. The integration of a tactile sensing component and a neuromorphic element is essential to emulate the tactile perception of a biological system. Kim et al. conducted landmark research concerning the artificial tactile system. They integrated clusters of pressure sensors providing a wide range of pressure information, ring oscillators enabling the conversion of pressure information into the electrical pulses, and a synaptic transistor for encoding information about external stimulus (Fig. 12c). The artificial afferent was integrated with a biological efferent nerve in discoid cockroaches to create a hybrid reflex arc. The pressure stimuli collected by the artificial afferent nerve were converted into a postsynaptic current of the synaptic transistors and amplified to control the biological muscles, which resulted in the contraction of the cockroach leg and the contraction force was dependent on the pressure intensities [41]. This proves that the artificial tactile system is a promising technology for potential applications in neurorobotics and neuroprosthetics.

Artificial nerves and artificial reflex arcs hold great promise in advancing medical technology and improving the quality of life for individuals with various disabilities [186–189]. Qu and the colleagues constructed an artificial corneal reflex arc containing a vibration sensor oscillation circuit to convert mechanical stimuli into electrical pulses, a zinc tin oxide (ZTO)-based artificial synapse to transfer and integrate the information, an amplifier circuit to output desired voltage to operate PEDOT:PSS-based actuator, which implement mechanical and light information coding, information processing and the regulation of transmitted light [186]. As research progresses, the integration of these systems with biological components may ultimately lead to innovative treatments and enhancements in human capabilities.

The distribution density of contact and pressure points on the skin surface and the corresponding sensory area of the cerebral cortex are directly correlated with the sensitivity of this part to touch pressure sensation. The most precise mechanoreceptors have a resolution at the level of 0.5 mm. It is therefore essential to develop artificial tactile sensors with high position sensitivity to realize applications that involve special operations or dangerous work, such as bomb disposal.

In summary, the rapid advancement in organic materials and processing techniques is enabling great progress in the use of organic neuromorphic devices as fundamental building blocks in artificial perception systems. A single artificial system based on the electrolyte-gated organic synaptic transistors can emulate the basic functions of the visual, auditory, olfactory, gustatory, and tactile systems. It is important to note that biological perception is a complex process, which is achieved through the combined actions of multiple perception systems rather than a single one. Our eyes allow us to recognize the shape of an object, our skin lets us perceive its temperature and texture, our ears enable us to perceive its compactness when tapping on it, and finally, our brain receives a comprehensive information set that enables our precise thinking and decision-making. It is therefore clear that integrating neuromorphic platforms with multiple perception functions is of great importance for the development of brain–computer interfaces and the applications in artificial intelligence.

### Artificial Neurons

In the previously described artificial perception systems, EGOSTs were primarily used as hardware-based implementations to mimic the synaptic functions in different perception systems, enabling efficient brain-inspired computing. Neurolink, a company founded by Elon Musk, has developed an implantable device comprising a microelectrode array with 3072 electrodes distributed across 96 threads for targeting specific brain regions to be used as brain–machine interfaces [190]. Directly interfacing electronics with biological systems sharing similar biocomputational paradigm offers significant potential for restoring sensory and motor functions, as well treating neurological disorders [191]. In general, the integration of neuromorphic electronics with biology requires artificial synapses that can interface with biological systems and operate in electrophysiological fluid environments [192]. Conventional electronics used for mimicking neuronal behavior are unsuitable for handling the diversity of biosignals and operating in the real biological environment due to their bulky biomimetic circuits and instability in moisture [193].

As stated in the third scenario, the electrolyte-gated organic synaptic transistors unquestionably exhibit outstanding synaptic plasticity, analogous to biology, with low power consumption, multiple conductance, tunable retention time and linearity in weight update. The soft nature of the organic channel and its ability to directly interact with ions in aqueous solution make it an ideal candidate for developing artificial neural circuits with ion/neurotransmitter-mediated spiking characteristics that resemble the spiking response of biological systems. In neurons, changes in ion concentration gradients between the intracellular and extracellular medium are associated to the excitation of action potentials. In the resting state, the extracellular space is dominated by sodium ions and the intracellular side is dominated by potassium ions. Once the cell membrane is depolarized, the voltage-gated Na^+^ channel opens, allowing the influx of Na^+^ until the membrane potential reaches close to the Na^+^ Nernst potential and a spike forms. Afterward, the repolarization occurs. Na^+^ channels close and K^+^ channels open, allowing the efflux of K^+^ across the cell membrane and restoring the membrane potential to the resting state.

**Fig. 13:**
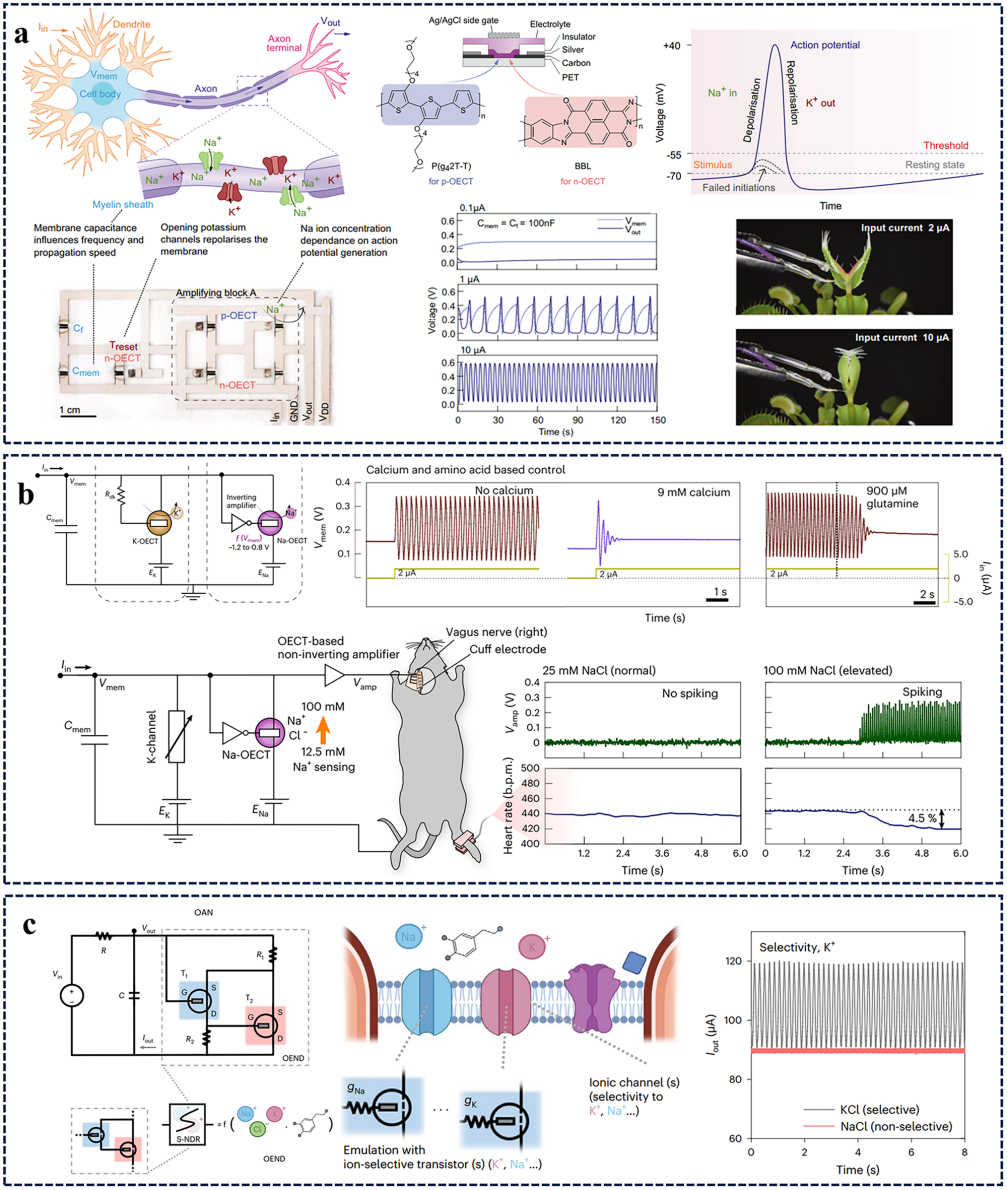
Bioinspired artificial neurons based on electrolyte-gated organic transistors. **a** Schematic illustration of the biological neuron and the organic electrochemical neuron and the output response of the spiking neuron to injected current and the integration of the spiking neuron to Venus Flytraps. Reproduced with permission [125]. Copyright 2022, Springer Nature. **b** Conductance-based organic electrochemical neuron (c-OECN). The c-OECN circuit with Na^+^- and K^+^-based OECTs and the modulation of spiking using Ca^2+^ and neurotransmitter glutamine. The integration of c-OECN circuit with the vagus nerve using an OECT-based amplifier and cuff electrodes and the electrophysiological response to different NaCl concentration. Reproduced with permission [87]. Copyright 2023, Springer Nature. **c** Circuit diagram of the organic artificial neuron (OAN) and its application in emulating the dynamics of biological ion channels. Reproduced with permission [194]. Copyright 2022, Springer Nature

Artificial neurons have recently been established to emulate the spiking process of nerve cells based on organic electrochemical transistors. Fabiano's group developed the first organic electrochemical neuron (OECN) with ion-modulated spiking based on all-printed complementary OECTs [125]. These OECTs were composed of a hole-transporting semiconductor (glycolated polythiophene-based p-type transistor) and an electron transporting poly(benzimidazobenzophenanthroline)-based n-type transistor which were used to build the amplifying block in the axon hillock circuit (Fig. 13a). The OECNs exhibited ionic concentration-dependent spiking. A low input current was insufficient to charge the membrane capacitors to reach the spiking threshold. Only for high input currents, spikes were initiated and their frequency increased with the input current, which resembled the operation of a nerve cell. They successfully integrated the OECN with a Venus flytrap (VFT) and used the spike output to stimulate the thigmonastic response of the VFT. A high spike frequency induced the release of Ca^2+^ in the cytosol, enabling the closure of the VFT. Harikesh et al. then presented another possibility: an all-printed complementary OECNs consisting of p-type and n-type OECTs according to the ion-tunable antiambipolar of BBL-based OECTs, which resulted from the reduction in the channel conductivity at high electrochemical doping. This led to the development of a conductance-based organic electrochemical neuron (c-OECN) [87]. The p-type OECT is dependent on Na^+^ concentration, stimulating action potential generation. The latter is responsive to K^+^, opening potassium channels and repolarizing the membrane. OECNs exhibit several neuronal characteristics when integrated with complementary OECNs. They are responsive to a wide range of input currents (0.1–10 µA), resulting in frequency modulation of over 450%. These organic electrochemical neurons were controlled using secondary ions such as Ca^2^⁺ and ammonium-based cations of glutamine/GABA/dopamine, as they can affect the hydrogen bonding interactions with the polymer. Furthermore, they coupled the c-OECN with the vagus nerve of a mouse using a cuff electrode and observed a reduction in the heart rate in response to an increased Na^+^ concentration (Fig. 13b).

In addition, the specificity of ion channels in biological membranes governing the reflux of ions in medium is of fundamental importance for neuronal signaling. This is because the channelopathies caused by the dysregulation of ion reflux can result in serious pathological conditions such as cystic fibrosis and myotonia congenita [195]. Gkoupidenis’s group has developed an organic artificial neuron (OAN) based on OECTs. PEDOT:PSS was as the active material, with ionophore-based selective membranes inserted between the organic channel and electrolyte to realize the selective processing of the ionic carriers (Na^+^ or K^+^) (Fig. 13c) [194]. The incorporation of a K^+^-selective membrane enabled the OAN to show oscillations with KCl electrolyte and constant behavior with NaCl electrolyte.

In summary, artificial neurons based on electrochemical transistors successfully replicate most neural features and will undoubtedly integrate with biological systems to control their physiological behaviors. Meanwhile, the spiking properties can be easily regulated by changing the types or local concentrations of ions, biomolecules, or neurotransmitters in the aqueous environments. This, together with the unique capability to sense multiple chemicals, physical, biological signals, and simple structure, and easy fabrication, makes the EGOTs a promising candidate for the development of novel biointegrable event-based sensors for applications in health monitoring and brain–computer interfaces.

## Conclusions and Outlook

Overall, EGOTs are suitable candidates as synaptic transistors for emulating the synaptic plasticity of biological systems. They can emulate both short-term and long-term plasticity with multiple conductance states and tunable retention. The soft nature of the organic materials, low voltage, as well as the operation in the same liquid environment as biological entities makes the synaptic transistor ideal for low power consumption and convenient communication with biological systems. Additionally, they exhibited tremendous potential in emulating neural perception and facile interfacing with biology for modulating spiking dynamics.

Significant progress have been made in the development of high-performance organic semiconductors and transistors. However, there are still some challenges to be overcome, including the development of organic semiconductors, multi-mode integration, biocompatibility, long-term durability, etc. In terms of organic semiconductors, the long-standing stable operation in aqueous medium is required to assemble the physiological functioning of biological neurons. However, the organic semiconductors typically exhibit poor stability in moisture due to the disruption of molecular conformation induced by swelling or degradation, particularly in n-type organic semiconductors. Furthermore, there is a trade-off between high carrier mobility for fast responses and low conductivity for low power consumption. A well-aligned crystalline structure accounts for high charge carrier mobility but hinders ion transport. Therefore, optimal design of the organic semiconductors is of great importance to concurrently realize the fast response matching with biological systems and low energy consumption. Furthermore, the sensitivity of organic materials to conventional photoresists represents a significant challenge in the patterning of organic materials for large-scale integration and miniaturization. This underscores the need to develop of novel orthogonal photoresists and processing techniques.

In terms of the working mechanism, devices relying on ion migration are normally more efficient than that of rely on electrochemical (redox) methods or charge trapping. However, strong shielding effect in an aqueous environment severely limits interionic interactions, preventing the emulation of synaptic and memory functions in fluidic-based systems. Meanwhile, parasitic (electro)-chemical reactions should be minimized in electrolyte in order to avoid the degradation of the device performance and introduction of toxic by-products to the living organisms in biointerfacing applications.

The disparity in interfacial messenger modalities is the other major issue in hindering the integration of the neuromorphic device with biological systems. The information encoding in the biological process is a complex process. It involves specific recognition and transmission of messengers including various ions, biochemicals, proteins, and nucleic acids. In contrast, the neuromorphic device encodes information using electrical signals. Meanwhile, the current artificial perception systems based on organic transistors are only capable of executing single perception tasks. Electrical devices are generally sensitive to only one species such as ions or electroactive biomolecules such as dopamine. In contrast, the human perception system is a multi-functional system requiring the collaboration of multiple modes of perception and its functioning in complex electrophysiological environments where also non-electroactive transmitters play a critical role in controlling the biological activities. By effectively addressing feature extraction, data alignment, and information fusion, multimodal learning provides a robust framework for interpreting complex data and delivering richer insights that single-modality approaches may miss. As the field continues to evolve, advancements in neural network architectures and training methods are likely to further enhance the capabilities of multimodal systems.

Furthermore, the majority of works employs redox reactions between targets and H_2_O_2_ to emulate the neurotransmitter-modulated electrophysiological activities. However, there is a lack of research focusing on the specific and selective interaction of the neuromorphic device with the neurotransmitter target, which could be achieved by employing antibodies, aptamers, or enzyme. Furthermore, it is crucial to develop flexible and stretchable neuromorphic device to achieve better biocompatibility in interfacing biology and the future implantable applications. Interfacing biological neural networks with neuromorphic devices is an important area for further investigation, as it could lead to a deeper understanding, restoration and augmentation of biological neurons. This requires the devices with low modulus to alleviate the immune response and controllable operation lifetime to be appropriate to the demanded duration. Therefore, developing hydrogel-based or biodegradable EGOSTs with controllable stability could be the direction of further exploration.

The development of bioinspired organic synaptic transistors is still in its infancy. To advance their performance and application in brain–computer interfaces, researchers from different fields must collaborate including materials scientists, biologists, chemists, electrical engineers, and computer scientists.
